# SSGA and MSGA: two seed-growing algorithms for constructing collaborative subnetworks

**DOI:** 10.1038/s41598-017-01556-z

**Published:** 2017-05-03

**Authors:** Xiaohui Ji, Su Chen, Jun Cheng Li, Wenping Deng, Zhigang Wei, Hairong Wei

**Affiliations:** 1 College of Information and Computer Engineering, Northeast Forestry University, Harbin, Heilongjiang 150040 P.R. China; 2 State Key Lab of Forest Genetics and Breeding, Northeast Forestry University, Harbin, Heilongjiang 150040 P.R. China; 3 Guangdong Key Laboratory for Innovative Development and Utilization of Forest Plant Germplasm, South China Agricultural University, Guangzhou, 510642 P.R. China; 4 School of Forest Resources and Environmental Science, Michigan Technological University, Houghton, MI 49931 USA; 5 Department of Computer Science, Michigan Technological University, Houghton, MI 49931 USA; 6 Life Science and Technology Institute, Michigan Technological University, Houghton, MI 49931 USA

## Abstract

The establishment of a collaborative network of transcription factors (TFs) followed by decomposition and then construction of subnetworks is an effective way to obtain sets of collaborative TFs; each set controls a biological process or a complex trait. We previously developed eight gene association methods for genome-wide coexpression analysis between each TF and all other genomic genes and ﻿then﻿ constructing collaborative networks of TFs but only one algorithm, called Triple-Link Algorithm, for building collaborative subnetworks. In this study, we developed two more algorithms, Single Seed-Growing Algorithm (SSGA) and Multi-Seed Growing Algorithm (MSGA), for building collaborative subnetworks of TFs by identifying the fully-linked triple-node seeds from a decomposed collaborative network and then growing them into subnetworks with two different strategies. The subnetworks built from the three algorithms described above were comparatively appraised in terms of both functional cohesion and intra-subnetwork association strengths versus inter-subnetwork association strengths. We concluded that SSGA and MSGA, which performed more systemic comparisons and analyses of edge weights and network connectivity during subnetwork construction processes, yielded more functional and cohesive subnetworks than Triple-Link Algorithm. Together, these three algorithms provide alternate approaches for acquiring subnetworks of collaborative TFs. We also presented a framework to outline how to use these three algorithms to obtain collaborative TF sets governing biological processes or complex traits.

## Introduction

We proposed the concept of collaborative network, which was originally called shared coexpression connectivity matrix (SCCM)^1^ rather than collaborative network because the whole collaborative network could be represented by a sparse symmetric matrix, with both dimensions to be the same transcription factors (TFs) and each intersection to be the shared number of coexpressed genes between two corresponding TFs, one in each dimension. The collaborative network depicts an interesting phenomenon that multiple groups of collaborative transcription factors (TFs) can be identified from gene profiles, and within each group, TFs act cooperatively to accomplish a task^1^, for example, collaboratively regulating a biological process like human pluripotency maintenance, *Arabidopsis* root cap maturity, and lignocellulosic biosynthesis. The TFs can be broadly defined as all regulatory genes including DNA-binding proteins, transcription activators and repressors, and chromatin modulators in a species. A collaborative network differs from a co-expressed network in that a collaborative network allows the transcription factors with roughly similar ‘behavior’ in expression profiles to be defined as “coordinated” and linked with an edge in the collaborative network. Similar behavior, in this case, can be interpreted as similar trends, peaks and nadirs in the full or partial expression profiles. To build a collaborative network of all transcription factors in the form of SCCM matrix, we first perform co-expression analysis between each *TF*_*i*_ and all genomic genes with one of correlation-based methods^2^, where ***i*** = (**1**, **2**, **3**, **…** ***n***) ***and n*** is the number of all TFs, and then sort the output by p-values, and apply a cut-off with a threshold to obtain the top k most tightly coexpressed genes to *TF*_*i*_, where k = 50, 100 or 150. The correlation-based methods can be eight gene association methods^2^ which include Spearman rank correlation, Weighted Rank Correlation, Kendall rank correlation, Hoeffding’s D measure, Theil-Sen, Rank Theil-Sen, Distance Covariance and Pearson. Secondarily, we calculate the number of common co-expressed genes between the top k co-expressed genomic genes to *TF*_*i*_ and the top k co-expressed genes to *TF*_*j*_, where ***j*** = (**1**, **2**, **3**, **…** ***n***). The shared number of co-expressed genes is the added to the intersection of *TF*_*i*_ and *TF*_*j*_ in SCCM matrix to represent the collaboration degree between *TF*_*i*_ and *TF*_*j*_. The significance of constructing a collaborative network lies in that the decomposition of such a network followed by reconstruction of internally well-connected gene clusters can result in multiple subnetworks of TFs, as displayed in our previous study^1, 2^, each subnetwork contains TFs that are collaborated to regulate or govern a biological process or a complex trait.

In contrast to the eight gene association methods for construction of collaborative networks (shared co-expression connectivity matrix)^2^, there is currently only one algorithm called Triple-Link Algorithm^2^, which can be used to build multiple collaborative subnetworks of TFs after a collaborative network is decomposed. Triple-Link Algorithm constructs collaborative subnetworks in three steps. First, identification of two nodes (TFs) with a maximal edge weight from the decomposed collaborative network. These two nodes are used as an initial seed. Secondly, adding a third node into the seed if it has two significant edges with two intra-seed nodes, with edge weights exceeding *n*_*e*_ = *μ* + *θδ*, where *μ* and *δ* are the mean and the standard deviation of non-zero edge weights in the whole collaborative network and *θ* = (*θ*_1_, *θ*_2_) ⊂ (0.5~1.0, 1.0~1.5). Thirdly, when the number of nodes within the growing seed exceeds three, a candidate node can be added into the growing seed if it has at least three significant edges with the intra-seed nodes; the three weights must exceed *n*_*e*_ = *μ* + *θδ*, where *θ* = (*θ*_1_, *θ*_2_, *θ*_3_) ⊂ (0.5~1.0, 1.0~1.5, 1.5~2.5) respectively. The three *θ*_1_, *θ*_2_, *θ*_3_ are corresponding to three differential thresholds that were used to determine if the candidate should be joined, with *θ*_1_ to be the least and *θ*_3_ to be the most stringent one. After that, the third step is executed repeatedly until no more candidate nodes satisfy the criteria of three significant edges. Then, a subnetwork is produced. The nodes within the built subnetwork are removed from the whole collaborative network before the above procedures are re-called to build the next subnetwork.

**Figure 1:**
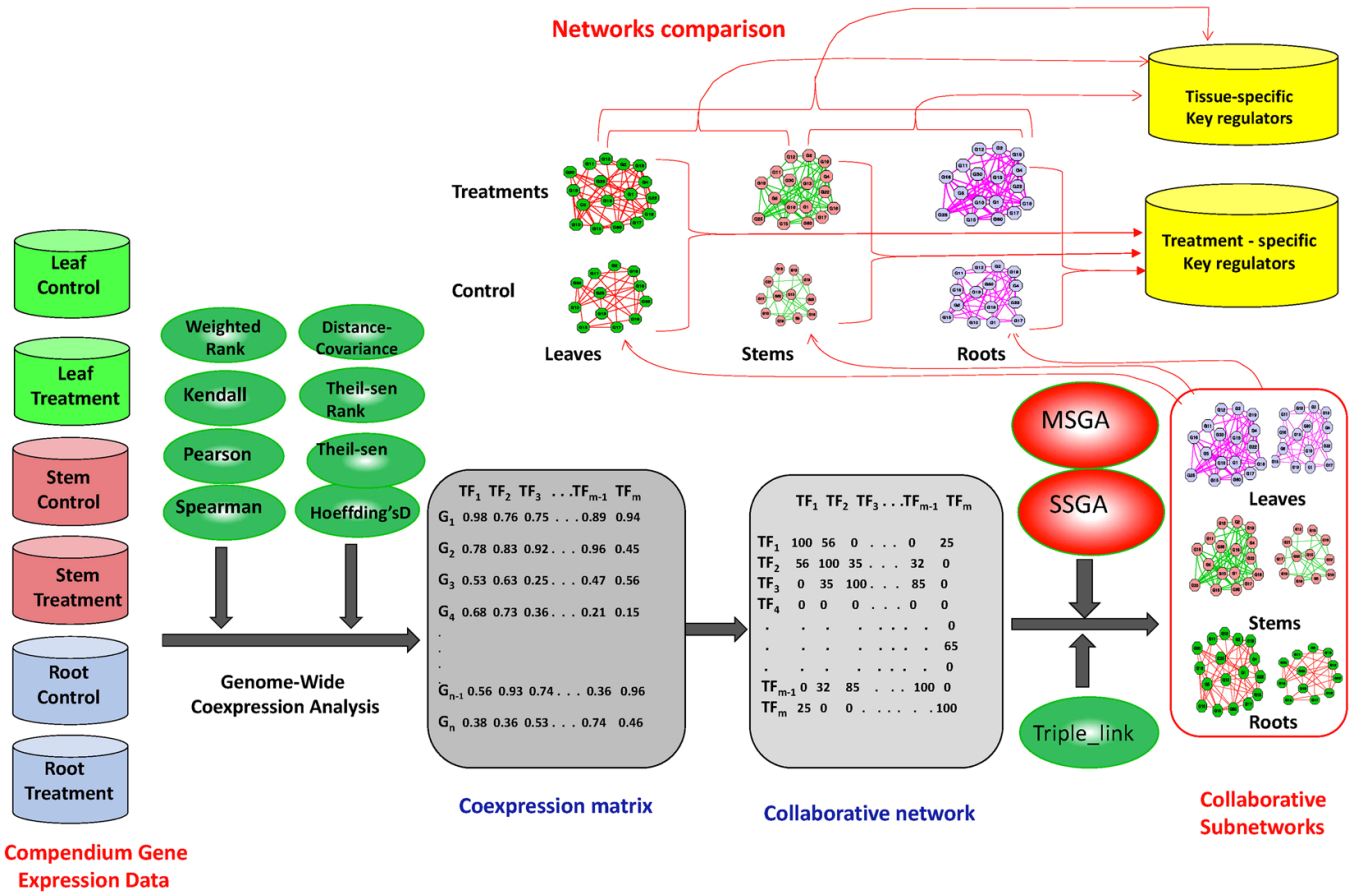
The illustration of a framework in which SSGA and MSGA are used to construct collaborative regulatory gene subnetworks, each governing a biological process or a complex trait. Multiple collaborative subnetworks can be constructed using data from different cell/tissue types or treatment conditions, and the resulting subnetworks can be compared for pattern recognition of key regulatory gene sets for specific treatment conditions or tissue/cell types.

As stated above, the Triple-Link Algorithm requires exact three significant links for each candidate node to be joined. Since this requirement is, to some degree, arbitrary, we have been deliberating about the existence of more efficient and accurate algorithms. In this study, we developed two novel algorithms, Single Seed-Growing Algorithm (SSGA) and Multi-Seed Growing Algorithm (MSGA), for building collaborative subnetworks based on two different schemes. The collaborative subnetworks built with these two new algorithms were carefully compared with those built using the Triple-Link Algorithm. The results indicate that the two new algorithms are more efficient and accurate than the Triple-Link Algorithm, and these novel algorithms can therefore serve as alternative approaches for building multiple collaborative subnetworks. We also presented a framework outlining where these three algorithms are suited for discovery of collaborative TF sets governing biological processes or complex traits. The use of eight gene association methods to build collaborative networks, followed by three alternative algorithms to build subnetworks of collaborative TFs, enables us to identify multiple TF sets; each regulates a biological process or a complex trait. A framework, as demonstrated in Fig. 1, is proposed to outline the potential uses of these methods and algorithms for discovering novel biological knowledge from high-throughput gene expression data in modern genomics research.

## Results

### Development of two algorithms for constructing subnetworks of highly collaborative TFs from a decomposed collaborative network through seed-growing approaches

We first built a collaborative network of all TFs using the Spearman rank correlation, which has a high accuracy in associating functionally collaborative TFs as evidenced in our earlier publications^1, 2^. After we obtained the collaborative networks (Supplementary Figure 1), we decomposed it and saved nodes (TFs) and all edges (coordinated relationships). We then used the two seed-growing algorithms SSGA and MSGA, as described in Materials and Methods, to acquire the subnetworks of collaborative TFs. Briefly, the basic principle underlying SSGA is that the three completely linked nodes with the maximal sum of edge weights among all triple-node was identified and used as a seed for growing a subnetwork, during which external nodes were incorporated into the growing seed gradually based on some criteria that include the certain number of authentic edges (﻿edge weight > S, see Methods)﻿ and edge weights between each external node and the intra-seed nodes. Seed growing was terminated when there was no external node that met the criteria. When a seed grew into a subnetwork, SSGA saved all nodes in the subnetwork while removing these nodes from the whole collaborative network. The algorithm then identified a new seed and repeated the above procedures to obtain a new subnetwork. This process was executed repeatedly until there was no seed available.

The second algorithm, MSGA, worked in a different tactic. MSGA first identified many seeds of three nodes and then each seed grew independently with a requirement that a complete graph (clique) was maintained after each node was added. The growing process was suspended when no additional nodes could be added to each growing seed while maintaining it as a complete graph, which is termed a seed core. MSGA then identified a new seed and grew it in the same way until it became a new seed core. When no more seed cores could be generated, MSGA began to merge the most closely associated seed cores in pairwise fashion based on specific criteria on the number of edges across two seed cores and edge weights. The two seed cores with the most closely coordination will be merged with a priority until no further seed core pairs could be merged. Finally, MSGA allowed the individual nodes that did not have full connections to all the nodes within a seed core to join, provided the number of connections to the intra-seed core and edge weights of these connections met certain criteria.

### Functional cohesion of TFs in the subnetworks derived from two seed-growing algorithms

We used SSGA and MSGA methods to decompose a collaborative network of 1622 TFs. This subnetwork was constructed in two steps as shown in Fig.1: (1) performing a genome-wide co-expression analysis between each TF and all genomic genes in *Arabidopsis thaliana* using Spearman correlation and a compendium data set we pooled from public repository; (2) building the collaborative network of all TFs as described earlier^1^. The collaborative network is represented by a symmetric matrix with both the horizontal and the vertical dimensions being 1622 TFs. Each value (0~100) in the matrix represents the degree of coordination of two TFs in the corresponding row and column. After this matrix was decomposed into the edges, SSGA and MSGA initiated the subnetwork construction process; each subnetwork contains a group of highly coordinated TFs. The comparison of subnetworks derived from SSGA and MSGA was conducted with the subnetworks built with Triple-Link Algorithm^1^ as a comparison.

The subnetworks derived from both SSGA and MSGA showed functional cohesion. The TFs in Subnetworks 1 and 5 derived from SSGA are shown in Supplementary Table 1. It is obvious that the TFs present in Subnetwork 1 are primarily involved in root growth. Seven out of nine TFs in this subnetwork evidently regulate the root hairs or root development. The 108 microarray data sets used to construct the collaborative network were generated from *Arabidopsis* roots under the salt stress condition; therefore, it is plausible that the roots under salt stress are altered significantly. Subnetwork 5 contains 14 TFs, and there is evidence that 13 of them are involved in the root vascular system and secondary cell wall development (Supplementary Table 1); these include VND7^3^, VND4^3^, SND2^4^, ADOF2^5^, LBD18^6^, MYB46^7^, MYB52^7^, MYB103^7^, MYB20^7^, and MYB54^7^. The TFs in Subnetworks 2 and 4 derived from MSGA are shown in Supplementary Table 2. They largely overlap with Subnetworks 2 and 26 derived from SSGA. Thirteen of the 16 TFs in Subnetwork 2 derived from MSGA have a role in root tip growth and development and pluripotency maintenance. Surprisingly, the four TFs, SMB, FEZ, NAC015 (BRN1), and NAC070 (BRN2), present in this subnetwork have been demonstrated to function in the root tips to control stem cell pluripotency^8^. FEZ promotes periclinal, root cap-forming cell divisions while SMB represses stem cell-like divisions in root cap daughter cells via negatively regulating FEZ activity^9^. Subnetwork 4 regulates the cell cycle and root growth. In addition, Subnetworks 9, 10, and 18 derived from SSGA contain TFs that regulate root lateral organ and meristem development, root apical-basal and adaxial identity determination, and the drought/salt stress, respectively. Subnetworks 4 and 16 derived from MSGA regulate root growth and drought stress, respectively. All subnetworks derived from SSGA, MSGA and Triple-Link Algorithms are shown in Supplementary Table 3. Annotations were added to TFs in most of subnetworks especially those that are among the top of the subnetwork lists if there are functional annotations existing in the literature or databases. For a data set from a specific condition, the highly ranked subnetwork are generally more functional cohesive than lowly ranked.

### Comparison of collaborative subnetworks resulting from SSGA or MSGA with those from the Triple-Link Algorithm

**Figure 2:**
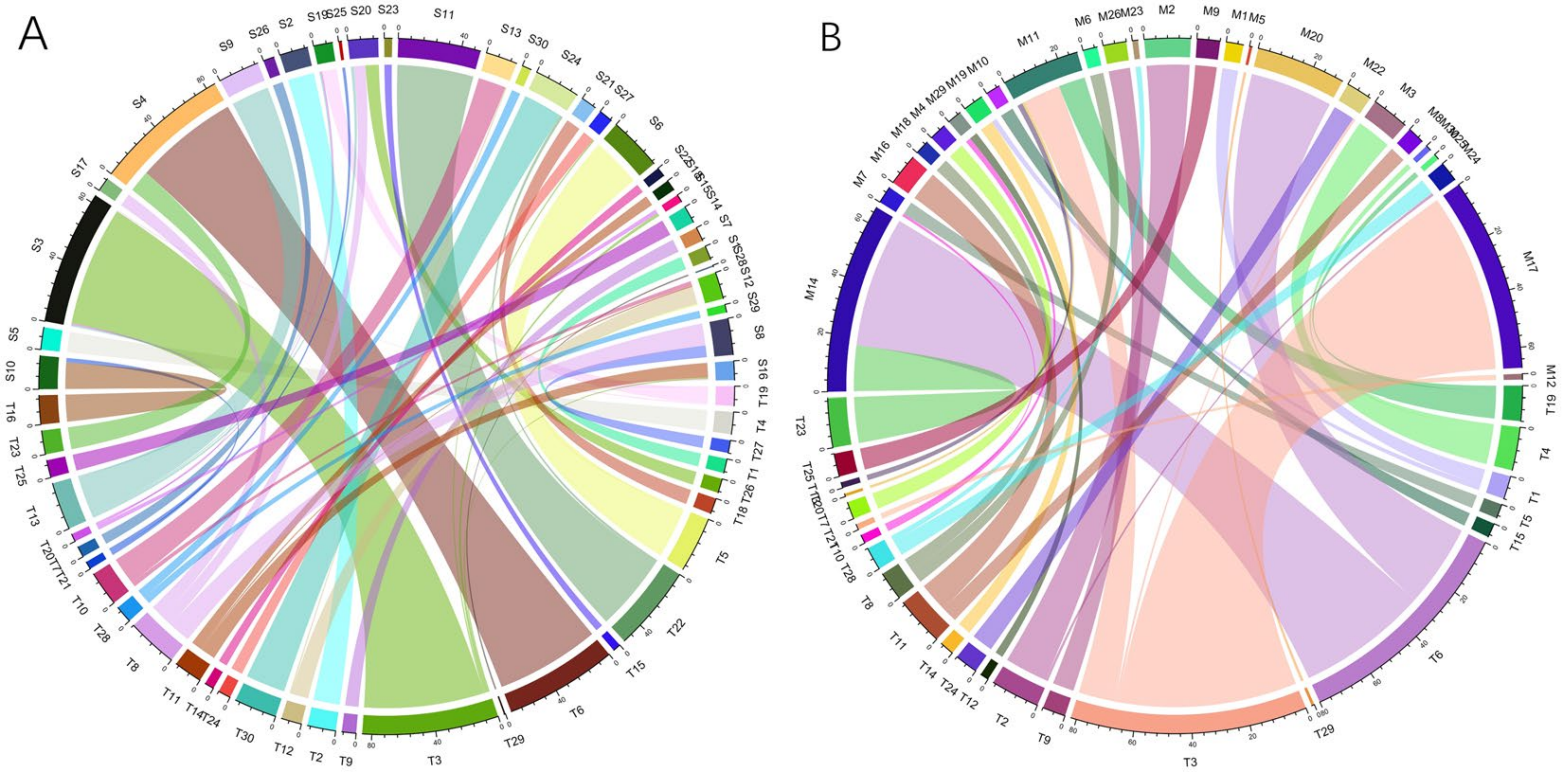
Comparison of collaborative subnetworks derived from SSGA or MSGA with those derived from Triple-Link Algorithm. (**A**) Comparison of the top 30 subnetworks derived from SSGA and Triple-Link Algorithm. The name of each subnetwork derived from SSGA is prefixed with “S,” whereas the name of each subnetwork derived from Triple-Link Algorithm is prefixed with “T”. Each curved line links the same gene in the two subnetworks derived from the two algorithms. (**B**) Comparison of the top 30 subnetworks derived from MSGA and Triple-Link Algorithm. The name of each subnetwork derived from MSGA is prefixed with “M,” whereas the name of each subnetwork derived from Triple-Link Algorithm is prefixed with “T”. Each curved line links the same gene in the two subnetworks derived from the two algorithms. Note that for the sake of clarity, only the common genes shared by two subnetworks generated with different algorithms are shown.

Although the majority of subnetworks resulting from SSGA or MSGA largely overlapped those that were generated by Triple-Link Algorithm, there are some subnetworks that are different, especially between the subnetworks of MSGA and those that were generated from two other algorithms. A comparison of the overlaps among the subnetworks built with the three algorithms is shown in Fig. 2A and B. For example, Subnetworks 25, 28, and 29 from SSGA only slightly overlapped to the subnetworks from Triple-Link Algorithm, whereas Subnetworks 15, 21, 27 and 28 of MSGA did not overlap to any of the subnetworks produced with Triple-Link Algorithm. Genes in Subnetwork 11 of Triple-Link Algorithm were split into Subnetworks 16 and 18 generated from SSGA. We calculated the intra-subnetwork average connections per node, and the intra-subnetwork average coordination strength measured with Spearman rhos of these three subnetworks. The average numbers of connections per node for Subnetworks 11 of Triple-Link, and 16 and 18 of SSGA were 0.538, 0.837, and 0.84, respectively (Supplementary Table 4) while the intra-subnetwork Spearman rhos for these three subnetworks in the same order were 0.416, 0.445 and 0.499, respectively (Supplementary Table 5). Subnetwork 4 of SSGA contains the genes from Subnetworks 6 and 23 of Triple-Link Algorithm. The average numbers of connections per node for Subnetworks 4 of SSGA, 6 and 23 of Triple-Link Algorithm were 0.851, 0.806, and 0.781, respectively (Supplementary Table 4) while the average intra-subnetwork Spearman rhos for these three subnetworks in the same sequence were 0.356, 0.349 and 0.318, respectively (Supplementary Table 5). The average number of outward connections per node and average coordination strength in subnetworks derived from MSGA were also augmented. For example, genes in Subnetwork 3 of Triple-Link Algorithm were split into Subnetworks 11 and 17 of MSGA. The average numbers of outward connections per node for Subnetworks 3 of Triple-Link Algorithm, and 11 and 17 of MSGA were 0.703, 0.883, and 0.808, respectively (Supplementary Table 4) while the averaged intra-subnetwork Spearman rhos for these three subnetworks in the same sequence were 0.407, 0.452, and 0.436, respectively (Supplementary Table 5). In another example, genes in Subnetworks 3 and 19 of Triple-Link Algorithm were unified by MSGA into Subnetwork 11. The average numbers of connections per node for Subnetworks 3 and 19 of Triple-Link Algorithm and 11 of MSGA were 0.703, 0.529 and 0.883 respectively (Supplementary Table 4) while the averaged intra-subnetwork Spearman rhos for these three subnetworks in the same sequence were 0.406, 0.428, and 0.452, respectively (Supplementary Table 5). More examples are provided in Supplementary Tables 4 and 5. We also showed two example figures that provides some clues regarding why the genes in Subnetwork 11 of Triple-Link Algorithm were split into two subnetworks (#16 and #18) of SSGA (Supplementary Figure 2) or and also two subnetworks (#16 and #8) of MSGA (Supplementary Figure 3). This is because in the Subnetwork 11 of Triple-Link Algorithm, the connections between those genes that showed up in Subnetwork 16 of SSGA and those genes that also showed in Subnetwork 18 of SSGA were relatively sparser as compared to connections within each of these two subnetworks. Similarly, in the Subnetwork 11 of Triple-Link Algorithm, the connections between those genes that showed up in Subnetwork 16 of MSGA and those genes that showed in Subnetwork 8 of MSGA were relatively sparser as compared to connections within each of these two subnetworks. For the above-mentioned reason, SSGA and MSGA both split Subnetwork 11 of Triple-Link Algorithm into two subnetworks. These results provide direct evidence that SSGA and MSGA increased not only the intra-subnetwork connections but also the intra-subnetwork association strength within their derived subnetworks as compared to Triple-Link Algorithm.

### Comparison of subnetworks derived from SSGA and MSGA and Triple-Link Algorithm in intra-subnetwork coordination strengths

**Figure 3:**
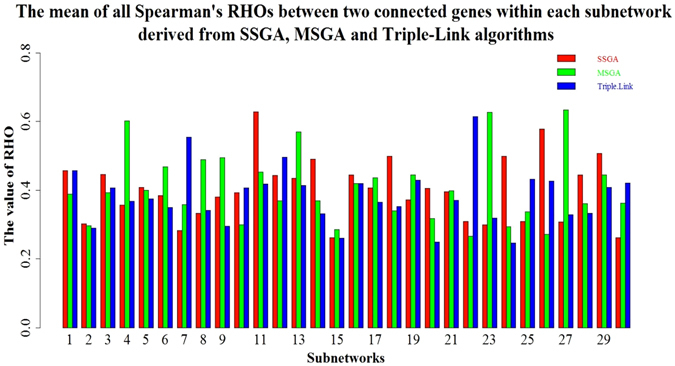
The mean of all Spearman’s rhos between two genes within each subnetwork derived from SSGA, MSGA and Triple-Link Algorithm. The TFs within a subnetwork with higher mean of rho values are more highly coordinated than those within a subnetwork with lower mean of rho values.

We further examined the intra-subnetwork coordination strengths of each subnetwork derived from the three algorithms. The intra-subnetwork coordination strengths were measured by cluster validity indices including Spearman rho, Davies Bouldin (DB)^10^, Davies Bouldin* (DB*)^11^, Xie-Beni (XB)^12^ and Xie-Beni* (XB*)^11^. The intra-subnetwork coordination strengths measured by Spearman rank rhos are shown in Fig. 3. Each bar is the average of all edges weights within a subnetwork. In general, the values of SSGA and MSGA are slightly higher than those of Triple-Link Algorithm. For example, 19 of 30 subnetworks produced by SSGA have higher average intra-subnetwork correlation coefficients than those produced by Triple-Link Algorithm, and 20 of 30 subnetworks derived from MSGA have higher average intra-subnetwork correlations than those derived from the Triple-Link Algorithm, suggesting that both SSGA and MSGA produce subnetworks in which regulatory genes have higher coordination. The reasons that the values of average intra-subnetwork rhos in Fig. 3 are not high in overall include: (1) we included all TFs for building collaborative networks; (2) the collaborative subnetworks include loosely coordinately TFs, as evidenced by the profiles shown in Supplementary Figure 4; and (3) TFs in higher hierarchic levels in general have lower correlation among themselves as compared to the co-regulated non-regulatory genes in lower hierarchic levels.

**Figure 4:**
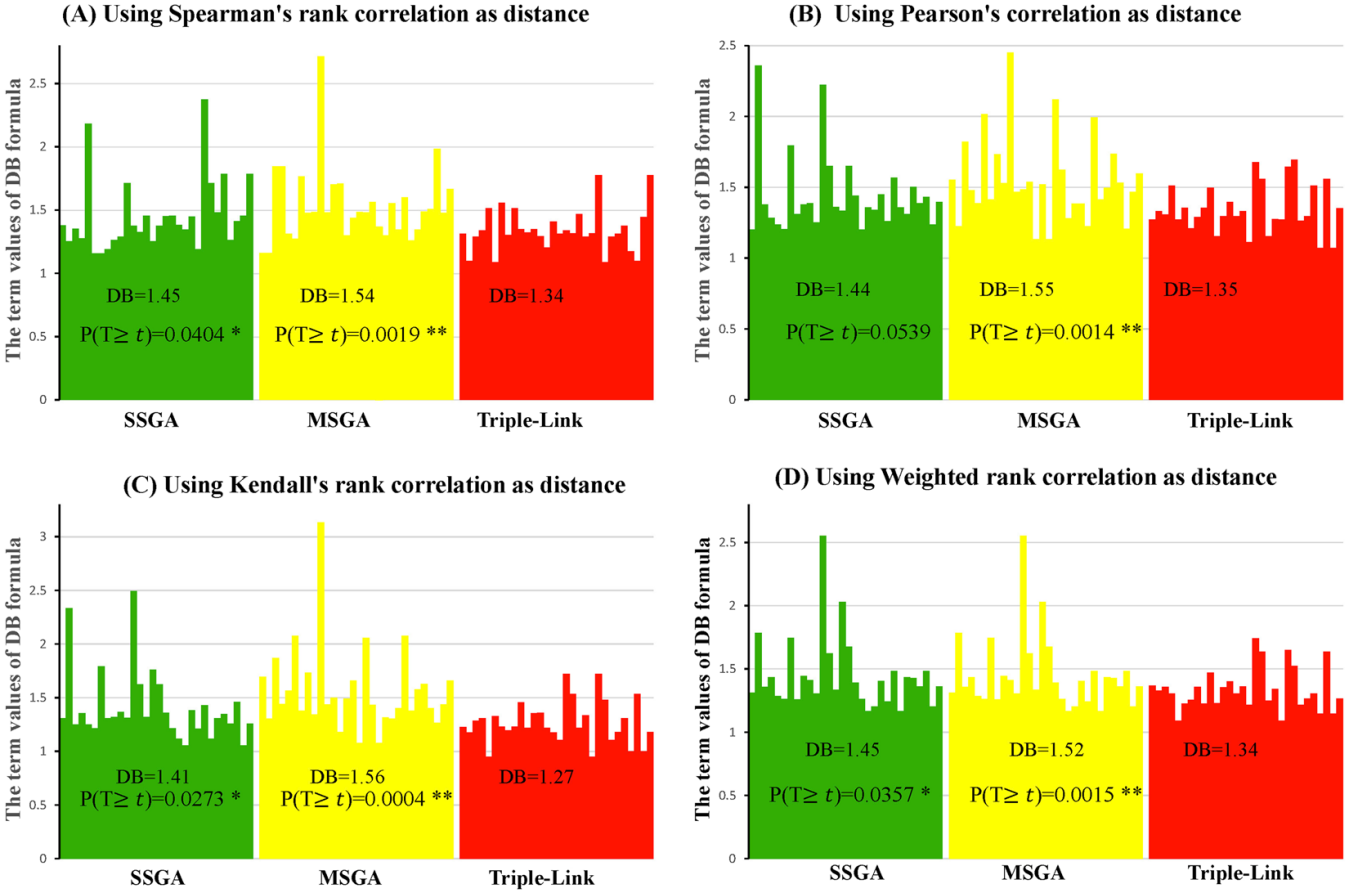
Davies-Bouldin (DB) indices for SSGA, MSGA and Triple-Link Algorithms, and the term scores of DB formula calculated for each of 30 subnetworks is represented by 30 bars. The distances used for calculating DB were based on Spearman’s rank correlation (**A**); Pearson correlation (**B**); Kendall’s rank correlation (**C**); Weighted rank correlation (**D**). In all the cases, the higher DB term scores or the DB indices, the tighter the internal coordination of derived subnetworks. The Student’s t-test (unpaired) was performed to examine the difference between DB’s term scores of 30 SSGA-/MSGA-derived subnetworks and DB’s 30 term scores of 30 Triple-Link Algorithm-derived subnetworks. The difference is significant if P(*T* ≥ ***t***) <**0.05**.

We next calculated Davies Bouldin (DB)^10^, Davies Bouldin* (DB*)^11^, Xie-Beni (XB)^12^ and Xie-Beni* (XB*)^11^ cluster validity indices. DB and DB* evaluate intra-subnetwork similarity versus inter-subnetwork differences, whereas XB and XB* define the minimum square distance between subnetwork centers and uses it for inter-subnetwork separation. The intra-network compactness is the mean square distance between each data object and its subnetwork center. The four types of DB indices calculated with Spearman, Pearson, Kendall and Weighted Rank correlation coefficients as distances are larger respectively in 22, 20, 20 and 18 subnetworks generated with SSGA compared to those produced by Triple-Link Algorithm, as shown in Fig. 4. In contrast, the four types of DB* indices calculated from Spearman, Pearson, Kendall and Weighted Rank correlation coefficients are larger in 23, 25, 23 and 22 subnetworks, respectively, derived from SSGA than those from Triple-Link Algorithm. With regard to the *Student*’s t-tests on the differences of DB indices between SSGA/MSGA and Triple-Link Algorithm, the differences of DB indices between SSGA and Triple-Link Algorithm were usually at significant levels while the differences between MSGA and Triple-Link Algorithm were always at extremely significant levels (Fig. 4A,B,C and D). In regard to the *Student*’s t-tests on the differences of DB indices between SSGA/MSGA and Triple-Link Algorithm, the differences of DB* indices between SSGA and Triple-Link Algorithm, and also between MSGA and Triple-Link Algorithm were always at extremely significant levels regardless which correlation method was used for calculating the DB* indices (Fig. 6A,B,C and D).

**Figure 5:**
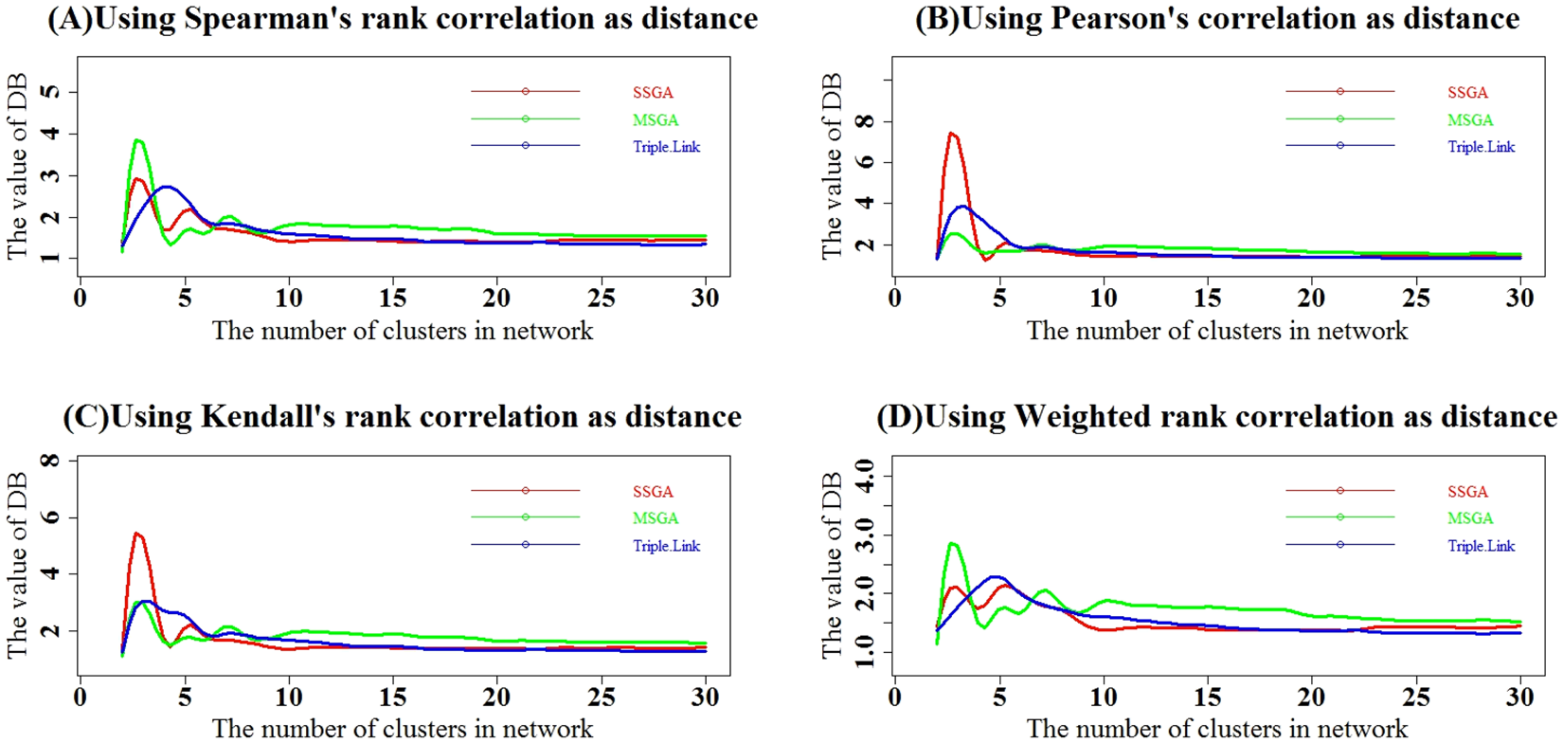
Comparison of SSGA, MSGA and Triple-Link Algorithm with the cumulative Davies-Bouldin (DB) indices. The cumulative DB indices of top *i* subnetworks derived from SSGA, MSGA and Triple-Link Algorithm where *i* = (2, …, 29, 30). (**A**) The distances used for calculating DB were based on Spearman’s rank correlation; (**B**). The distances used for calculating DB were based on Pearson correlation; (**C**). The distances used for calculating DB were based on Kendall’s rank correlation; (**D**). The distances used for calculating DB were based on Weighted Rank correlation. In all cases, a higher DB index of top *i* subnetworks reflects a more tightly averaged coordination of the top *i* subnetworks.

We also calculated the cumulative DB indices for top *i* subnetworks where *i* = 2, 3, 4, …, 30. As shown in Fig. 5, the four types of cumulative DB* indices calculated with Spearman, Pearson, Kendall and Weighted Rank correlation coefficients as distance suggest that SSGA had larger DB* values than Triple-Link Algorithm in the top seven subnetworks, whereas MSGA had approximately the same cumulative DB* values in the top 3, 4 or 5 subnetworks as compared to the Triple-Link Algorithm, and had slightly larger cumulative DB* values from the top 10 to top 22 subnetworks than either SSGA or the Triple-Link Algorithm. Cumulative DB* values of SSGA and MSGA were not different.

**Figure 6:**
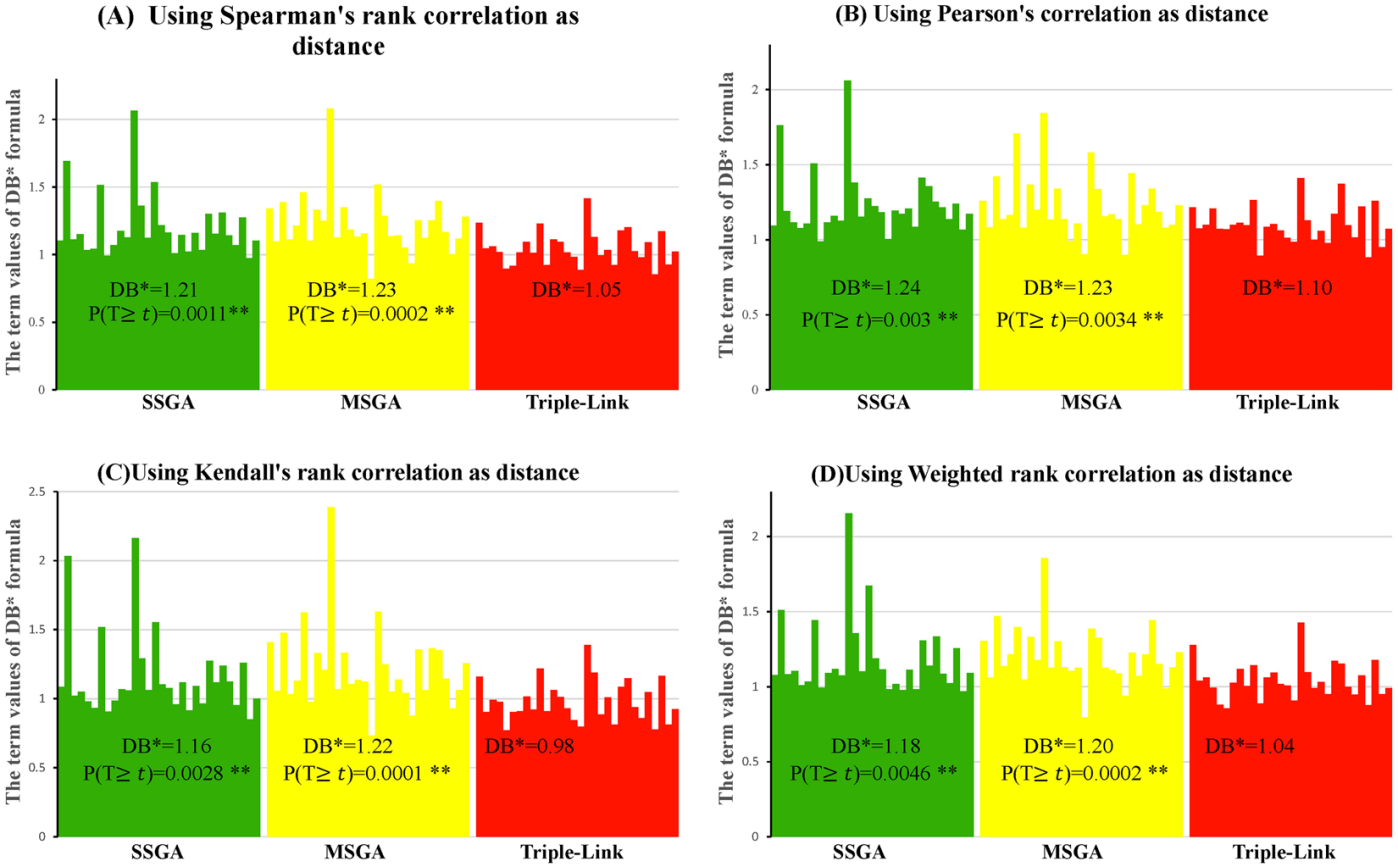
Davies-Bouldin* (DB*) indices for SSGA, MSGA and Triple-Link Algorithm, and the term scores of DB formula calculated for each of 30 subnetwork is represented by 30 bars. The distances used for calculating DB* were based on Spearman’s rank correlation (**A**); Pearson correlation (**B**); Kendall’s rank correlation (**C**); Weighted rank correlation (**D**). In all the cases, the higher DB* term scores or the DB* indices, the tighter the internal coordination of derived subnetworks. The Student’s t-test (unpaired) was performed to examine the difference between DB*’s term scores of 30 SSGA-/MSGA-derived subnetworks and DB*’s 30 term scores of 30 Triple-Link Algorithm-﻿derived subnetworks. The difference is significant if P(*T* ≥ ***t***) <**0.05**.

Similar to DB indices, the four types of DB* indices calculated with Spearman, Pearson, Kendall and Weighted Rank correlation coefficients as distance are larger in 24, 22, 23 and 23 SSGA-derived subnetworks, respectively, than in the corresponding Triple-Link–derived subnetwork (Fig. 6). In contrast, the four types of DB* indices calculated with Spearman, Pearson, Kendall and Weighted Rank correlation coefficients as distances were larger in 27, 24, 27 and 26 SSGA-derived subnetworks, respectively, than in the corresponding Triple-Link-derived subnetworks (Fig. 6).

**Figure 7:**
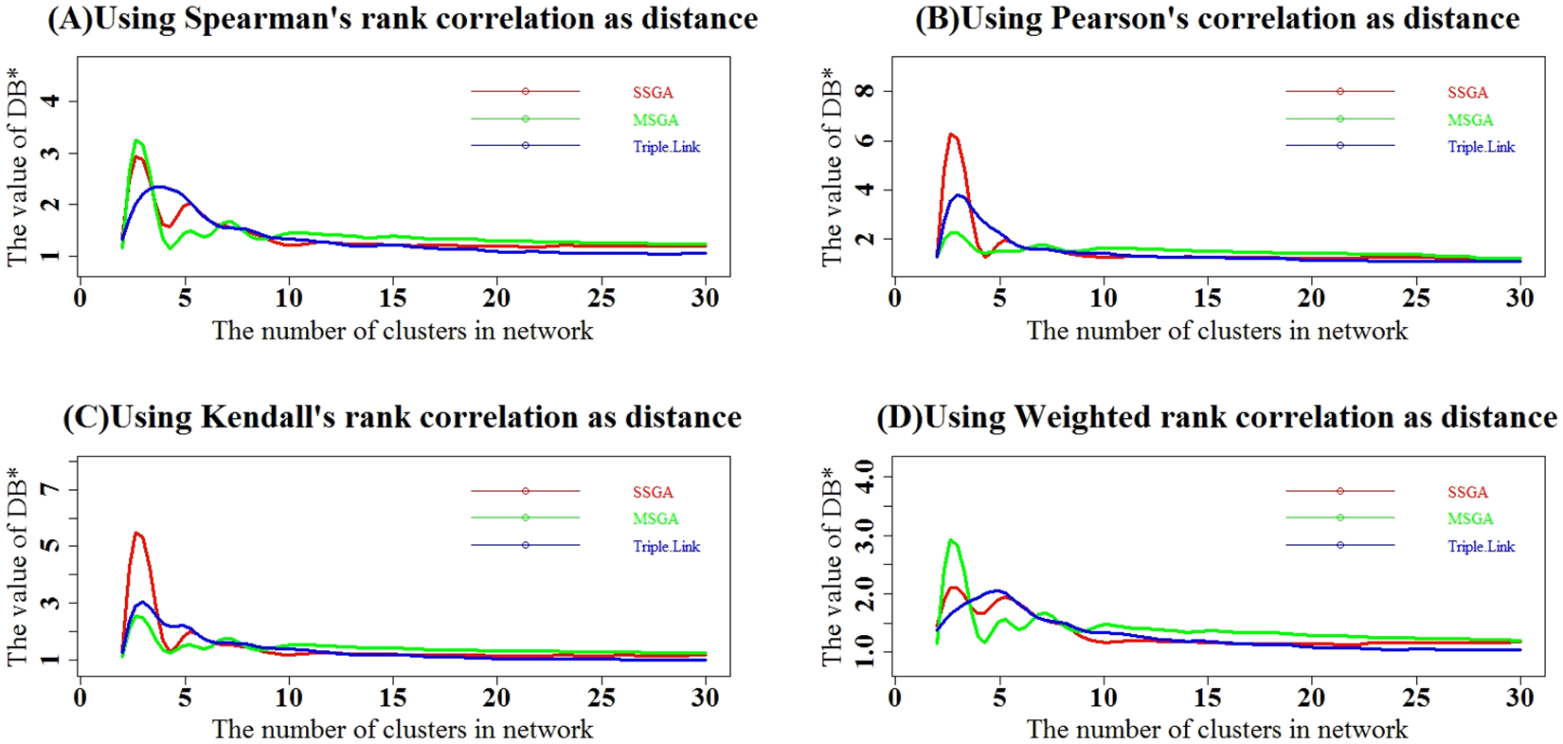
Comparison of subnetworks derived from SSGA, MSGA and Triple-Link Algorithm with DB* indices. The DB* indices of top *i* subnetworks derived from SSGA, MSGA and Triple-Link Algorithm where *i* = (2, …, 29, 30). (**A**) The distances were calculated based on Spearman rank correlation; (**B**). The distances were calculated based on Pearson product-moment correlation; (**C**). The distances were calculated based on Kendall’s rank correlation; (**D**). The distances were calculated based on Weighted rank correlation. In all the cases, the higher XE of top *i* subnetworks reflects a tighter average coordination of the top *i* subnetworks.

We also calculated the cumulative DB* indices for the top *i* subnetworks where *i* = 2, 3, 4, …, 30. As shown in Fig. 6, the four types of cumulative DB* indices calculated with Spearman, Pearson, Kendall and Weighted Rank correlation coefficients as distances suggest that SSGA had larger DB* values than the Triple-Link Algorithm in the top 3~7 subnetworks, whereas MSGA had approximately the same cumulative DB* values in the top five subnetworks compared to the Triple-Link Algorithm (Fig. 7). MSGA also had slightly larger cumulative DB* values in the top 10 to top 22 subnetworks than either SSGA or the Triple-Link Algorithm. Cumulative DB* values of SSGA and MSGA did not show distinct differences in the top 10 to 22 subnetworks (Fig. 7).

**Figure 8:**
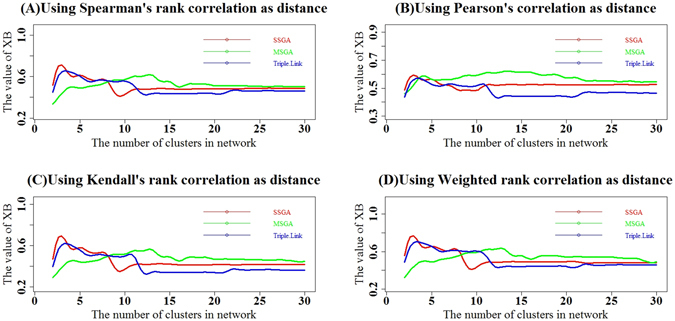
Comparison of subnetworks derived from SSGA, MSGA and Triple-Link Algorithm with XE indices. The XE indices of top *i* subnetworks derived from SSGA, MSGA and Triple-Link Algorithm where *i* = (2, …, 29, 30). The XE indices were calculated with Spearman rank correlation coefficients as distances (**A**), Pearson product-moment correlation (**B**), Kendall’s rank correlation (**C**) and Weighted rank correlation (**D**). In all the cases, a higher XE* index represents a greater degree of coordination in the top *i* subnetworks. In all the cases, a higher XE index represents a greater degree of coordination in the top *i* subnetworks.

We calculated the cumulative XE indices for the top *i* subnetworks where *i* = 2, 3, …, 30. As shown in Fig. 8, the four types of cumulative XE indices calculated with Spearman, Pearson, Kendall and Weighted Rank correlation coefficients as distance suggest that SSGA had slightly larger or approximately the same XE values compared with those for Triple-Link Algorithm in the top 7 subnetworks. MSGA-derived subnetworks had smaller cumulative XE values in the top 7 subnetworks compared to Triple-Link Algorithm (Fig. 8) but had slightly larger cumulative XE values from the top 10 to the top 30 subnetworks compared to either SSGA or Triple-Link Algorithm. The cumulative XE values of SSGA and MSGA did not show obvious differences in the top 10 to the top 30 subnetworks (Fig. 8).

**Figure 9:**
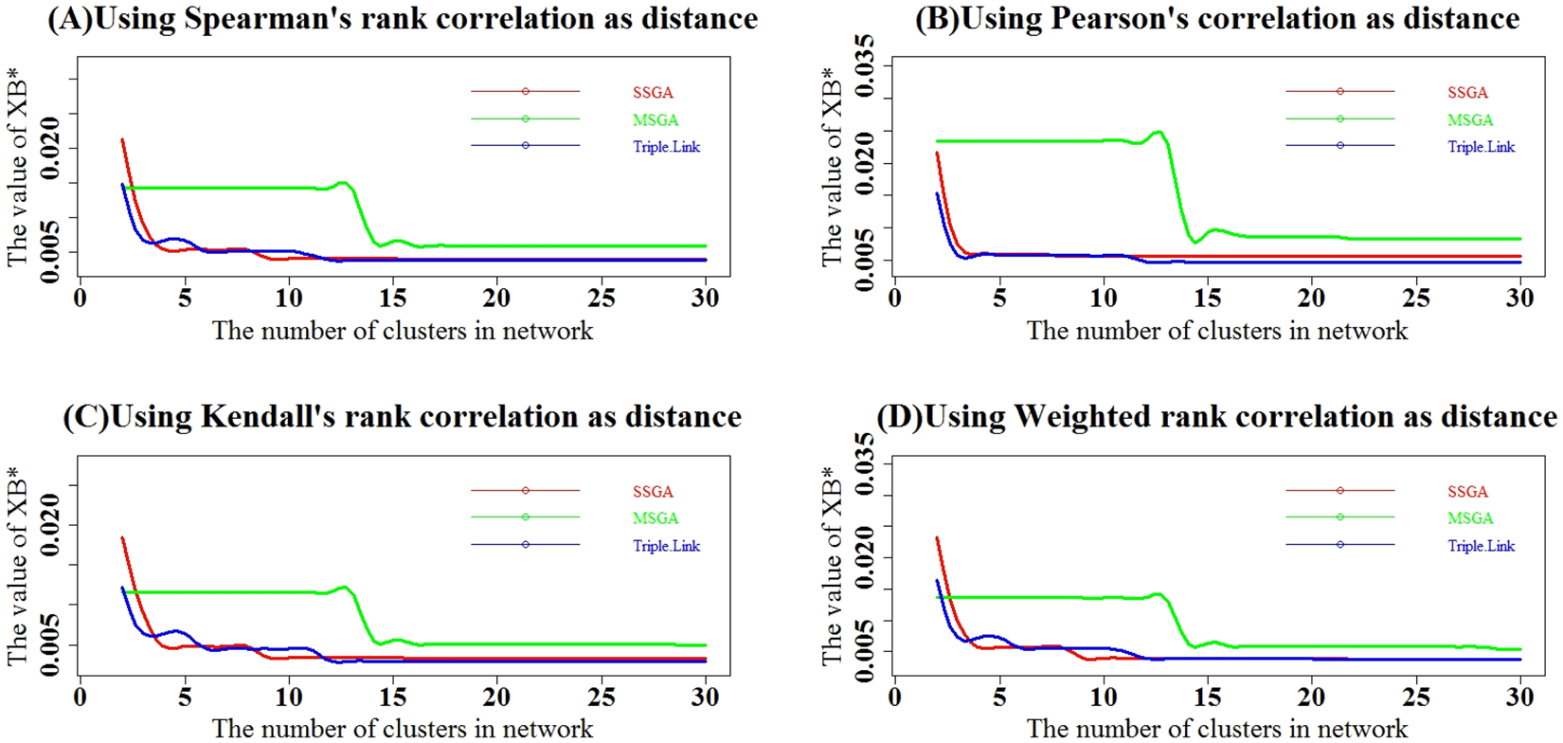
Comparison of subnetworks derived from SSGA, MSGA and Triple-Link Algorithm with XE* indices. The XE* indices of top *i* subnetworks derived from SSGA, MSGA and Triple-Link Algorithm where *i* = (2, …, 29, 30). The XE* indices were calculated with Spearman rank correlation coefficients as distances (**A**), Pearson product-moment correlation (**B**), Kendall’s rank correlation (**C**) and Weighted rank correlation (**D**). In all the cases, a higher XE* index represents a greater degree of coordination in the top *i* subnetworks.

We also calculated the cumulative XE* indices for top *i* subnetworks where *i* = 2, 3, …, 30. As shown in Fig. 9, the four types of cumulative XE* indices calculated with Spearman, Pearson, Kendall and Weighted Rank correlation coefficients as distance suggest that SSGA had a slightly larger or approximately the same XE* values as Triple-Link Algorithm in the top seven subnetworks, whereas MSGA had smaller cumulative XE* values in the top 3 subnetworks compared to Triple-Link Algorithm (Fig. 9). MSGA also had slightly larger cumulative XE* values from the top 4 to the top 30 subnetworks compared to either SSGA or Triple-Link Algorithm. The cumulative XE* values of SSGA and MSGA were not obviously different in the top 4 to the top 30 subnetworks (Fig. 9).

## Discussion

We developed two novel algorithms, SSGA and MSGA, for converting a collaborative network into multiple subnetworks through reconstruction, each collaborative subnetwork regulates a biological process or a complex trait. We have shown that the subnetworks derived from these two algorithms had functional cohesion and are biologically interpretable. The subnetworks derived from the two algorithms together with those from Triple-Link Algorithm are shown in Supplementary Table 3. Overall, SSGA yielded subnetworks that largely agree with those of the Triple-Link Algorithm from TF-Cluster package^1^, while MSGA produced the subnetworks that have slightly more discrepancy. In addition, the subnetworks derived from SSGA have comparable sizes to those obtained with the Triple-Link Algorithm, while subnetworks derived from MSGA have relatively smaller sizes. These differences are rooted in the different strategies employed in seed-growing procedures. In SSGA, a candidate node was added into a seed only if the number of connections and edge weights between this node and the seed exceeded the criteria; this is, to some extent, similar to the Triple-Link Algorithm. In MSGA, a candidate node was added into a seed only if it had a connection to every node in a seed in the early stage, though some candidates with incomplete connections to all intra-seed nodes were added at a later time. In this sense, MSGA is prone to emphasize full connections during the seed core growing stage. In addition to these descriptive discrepancies, we also summarized the performance of three algorithms in Supplementary Table 6.

### The comparison of the coordination strengths and connectivity within subnetworks derived from SSGA and MSGA with those from the Triple-Link Algorithm

In most circumstances, the subnetworks derived from SSGA and MSGA agree with those derived from Triple-Link Algorithm. Nevertheless, we found that SSGA and MSGA sometimes split or merged the subnetworks derived from the Triple-Link Algorithm. In this circumstance, SSGA and MSGA enriched the connections and also the coordination strengths in the split subnetworks or merged subnetworks as compared to original subnetwork(s) produced by Triple-Link Algorithm. The evidence of enrichment in connections and coordination strengths is shown in the results contained in Supplementary Figures 2 and 3 and Supplementary Tables 4 and 5, suggesting that SSGA and MSGA algorithms have great potential to produce the collaborative subnetworks in which TFs are more well-connected and have higher coordination than in those obtained with the Triple-Link Algorithm.

The cluster validity indices, DB, DB*, XE and XE*, (Figs 4, 5, 6, 7, 8 and 9) clearly indicate that SSGA and MSGA have some advantages over the Triple-Link Algorithm, for instance, the higher intra-subnetwork coordination and greater number of inter-subnetwork connections. Such a discrepancy entails SSGA and MSGA to be used as alternate and more effective approaches for constructing collaborative subnetworks. Results with DB, DB*, XE and XE* also indicate that SSGA tends to produce subnetworks with high intra-subnetwork coordination in the early construction stage while MSGA appears to produce subnetworks with high intra-subnetwork coordination in the middle stage of subnetwork construction (Fig. 7).

### The parameters used in SSGA and MSGA that can be tuned for obtaining expected results

In SSGA, we introduced a threshold parameter, θ, to build subnetworks of collaborative TFs. The θ represents the ratio of the number of authentic connections between a candidate node and the intra-seed nodes to the theoretic number of connections, and it was used to control seed growing. To determine the appropriate range of θ, we built collaborative networks with five different gene association methods that included Spearman rank, Pearson, Weighted rank, Kendall rank, and Hoeffding’s D methods as described earlier^2^. After that, the collaborative network was decomposed and the subnetworks were constructed with Triple-Link Algorithm. We calculated the θ and the results are shown in Supplementary Figure 5. We found the range of θ was almost exclusively larger than 0.5 but smaller than 1 in the subnetworks constructed. In this study, we set the default at θ = 0.6, but θ can actually be a choice between 0.55 and 0.75. If a higher θ is used, more edges with relatively smaller weights are excluded, and the resulting subnetworks are smaller and have higher intra-subnetwork coordination. Users can choose a θ from 0.55 to 0.75 or even higher to obtain subnetworks with different coordination strengths. In addition to θ, the parameter w, which represents the averaged edge weight between a candidate node and the intra-seed nodes, was used to entail the competitive ground where different candidate nodes can compete with each other for an opened position within the seed. When two or more candidate nodes have the same W value, SSGA compares which candidate can bring the edge distribution advantage after being added into a seed. The edge distribution advantage here refers to the more evenly distributed edges within the whole subnetwork. For MSGA, in addition to θ, there are also several parameters that can be tuned for obtaining different results. The first parameter is the *S*_*wd*_, which controls if two seed cores should be merged. The second is T, which controls how many individual nodes should be added to a merged or mature seed core. The selection of appropriate *S*_*wd*_ and T parameters can eventually affect the sizes of subnetworks. ﻿Moreover, there is a parameter α that is a percentage weight between edge weights and edge numbers for calculating both *S*_*wd*_ and T. The change of α may affect subnetwork topology.

### Data required for construction of collaborative network

Both the time-course data and non-time-course can be used to build collaborative networks. Time-course data with very small intervals from specific tissue type under a treatment condition may have more advantages as compared to non-time-course data because time course data may represent a continuous and synchronized process for capturing continued coordination. However, this does not mean that non-time-course data set is not qualified for being used for construction of collaborative networks. The compendium data set used in this study was pooled from six independent microarray experiments on *Arabidopsis* roots under salt treated condition, and is adequate for discovering many collaborative TFs. However, limited data under the control condition should be included in the compendium data because the control condition can be visualized as an initial point of an experimental treatment. Nevertheless, the proportion of control data in the whole compendium data set should not be overwhelming, otherwise the coordination of TFs may be attenuated and is less specific for the treated condition.

### The biological significance of collaborative network

Construction of collaborative subnetworks is untapped approach to identify coordinated TFs that govern the same biological process or pathway. Compared to markers-assistant approaches for identifying genes that control a trait, collaborative subnetwork construction is a more direct one. It may be more effective because it is based on the footprints of regulation events in gene expression profiles rather than the information encoded in DNA sequences, for instance, genome-wide gene association by SNPs. It is known that a majority of SNPs fall in non-coding regions and many of them are intergenic^13^. Even more incomprehensible, the affected genes are often up to 2 Mbps away from the associated SNP, and are not necessarily the closest genes to the SNP^14^. We have tried our Triple-Link Algorithm to multiple data sets, and many examples have been shown in our previous studies^1, 2^. Triple-Link Algorithm can identify multiple collaborative subnetworks with cohesive functions from just one data set. For example, the identification of 24 pluripotency maintenance genes from one compendium data set of human stem cells was accomplished at one stroke. Of these 24 genes, 17 were identified by biologists in many years of struggle with multi-million dollar budget.

Figure 1 is used to illustrate that we could construct various subnetworks using the algorithms and framework we proposed as more and more gene expression data are deposited into public repositories. For example, (1) subnetworks constructed with the data sets from the same tissue type but under different environmental conditions enable us to investigate how TFs collaborate differentially in response to different environment cues; (2) subnetworks constructed with the data sets from multiple tissue types but under the same environmental condition (e.g. drought) enable us to investigate how TFs collaborate differently in various tissue types in response to the same environmental cue. We believe the algorithms we developed will find their way into a wide range of applications for novel biological knowledge discovery.

## Materials and Methods

### Construction of collaborative network of transcription factors

The salt stress compendium microarray data set comprises 108 microarrays from 6 experiments that have the accession identifiers of GSE7673, GSE7639, GSE7641, GSE7642, GSE8787 and GSE5623 in the NCBI GEO database (http://www.ncbi.nlm.nih.gov/geo/). More details about this compendium data and preprocessing process were described in our previous publication^1, 15^. Briefly, The original CEL files were downloaded and processed by the robust multi-array analysis (RMA) algorithm^16^ using the Bioconductor package. The collaborative network was constructed in two steps: (1) performing co-expression analysis between each of 1622 annotated TFs present in Affymetrix ATH1 matrix and all genomic genes using Spearman rank correlation method contained in the TF-Cluster Package^1^. The co-expressed genes of each TF were sorted by their p-values and the top 100 most tightly co-expressed genes to each TF were used to build collaborative network as described earlier^1^. Briefly, if the number of common genes between the top 100 most tightly coexpressed to the *TF* ( *i* ) and the top 100 genes most tightly coexpressed to the *TF* ( *j* ) is 35. Then, the 35, as the edge weight between TF(i) and TF(j) in coexpression network, is entered to the intersection of TF(i) and TF(j) in collaborative network, which can be represented by a symmetric matrix with both dimension to be all 1622 TFs. The collaborative network of all TFs built from this compendium data set was shown in Supplementary Figure 1 while the properties of this network were displayed in Supplementary Figures 6 and 7.

### Single seed-growing algorithm for construction of subnetworks of collaborative TFs

Step 1. We first decomposed the network to obtain all nodes (genes), edges and edges’ weights (strength of coordination), and calculate the average $(\bar{x})$ and standard variance (*σ*) of all the edges’ weights.

Step 2. Set up a threshold *S*, where $S=\bar{{\boldsymbol{x}}}+{\boldsymbol{\sigma }}$. The edges whose weights were larger than *S* were termed authentic edges.

Step 3. We selected three completely connected nodes as a seed. All the edges within the seed must be an authentic edge, and the sum of all three edge weights of this seed is maximal among all candidate seeds.

Step 4. Growing the seed into a subnetwork using the following procedures.

First, we evaluated all other candidate nodes for their authentic connections to the nodes within a seed, and select those that met the following criteria: (1) the ratio of the number of authentic connections between a candidate node and the intra-seed nodes to the theoretic connections (namely the number of intra-nodes) was larger or equal to theta (**θ**), where the **θ** is the threshold used to controlling the sizes of subnetworks. The value of **θ** ranges from 0.55–0.65, which was determined empirically based on the analyses of within-subnetwork connections decomposed from Triple-Link Algorithm outputs^1^. Note that θ is a variable that varies from time to time during decomposition process. (2) Then the weighted average weight, W [Eq. (1)], of authentic weights between each of selected candidate node and all intra-seed nodes will be calculated, and the candidate node with the maximum W was added into the seed.

1 $${\rm{W}}=\frac{{\sum }_{k=1}^{n}{x}_{k}^{2}}{{\sum }_{k=1}^{n}{x}_{k}}.$$

Where *x*_*k*_ is the weight of an authentic connection between a candidate node and an intra-seed node. n is the number of intra-seed nodes currently in the subnetwork. The intuition behind equation (1) is that we hope the sum of authentic connection between a candidate node and an intra-seed node is large, but we put more emphasis on larger ones.

However, if there are two or more candidate nodes that had equal values of W, we added each of them into the seed for some further evaluation. We calculated the coefficient of variation parameter K [Eq. (2)] of the growing-seed. The coefficient of variation is a measure of spread that describes the amount of variability relative to the mean. K can be used to evaluate if the distribution of nodes’ authentic connections within the growing seed were evenly and cohesively connected. The smaller K is an indicative of more cohesively and evenly connected the nodes within the growing seed. After evaluating all candidate nodes with the same W values, we formally added the one with the minimum K into the seed. However, if there were two or more selected candidate nodes that also had the same values of K, they were added into the seed at the same time.

2 $${\rm{K}}=\frac{{\rm{\sigma }}}{\bar{{\rm{c}}}}=\sqrt{\frac{1}{{\rm{N}}}\sum _{{\rm{i}}=1}^{{\rm{N}}}{({{\rm{c}}}_{{\rm{i}}}-\bar{{\rm{c}}})}^{2}}/\bar{{\rm{c}}}$$

Where *c*_*i*_ is the number of authentic connections of each node in the seed, N is the number of nodes in the seed, $\bar{c}$ is the average of nodes’ authentic connections in the seed.

Secondly, iterate Step 4 until there is no more nodes can be added into the seed, now the seed has matured and it contains a well-connected subnetwork.

Step 5. For each node within the subnetwork (or matured seed), we calculated the ratio of the number of authentic connections to the number of theoretic connections. If the ratios for all nodes are larger or equal to the threshold of θ, then we obtained a perfect subnetwork. If the node’s ratio is smaller than θ, we moved the node from the subnetwork into the candidate nodes, and repeated Step 5 until the ratios for all nodes in the subnetwork are larger or equal to the threshold θ.

Step 6. Repeat Steps 3 to 5 until all three-node candidate seeds were grown into subnetworks, each represents a set of TFs that are coordinated tightly.

### Multiple seed growing algorithm (MSGA) for construction of subnetworks of collaborative TFs

Steps 1–3. Same as those for SSGA.

Step 4. Grew the seed into a clique’s graph.

4.1 First, we selected a candidate node that met the following criteria: 1) there was one authentic edge between the selected node and each intra-seed node, 2) the selected node’s W value that was calculated based on the weights of all edges between the selected node and the intra-seed nodes is the largest among all candidate nodes. We then added the node into the seed.

4.2 Iteratively executed Step 4.1 until there are no candidate nodes that met the criteria aforementioned. The seed of this status quo was called–a seed core that represents a complete graph.

Step 5. Repeat Step 3 and Step 4 to obtain all seed possible cores.

Step 6. **Merging the seed cores with high connections:** Among all the seed cores, if the ratio of the number of authentic connections (n) between two seed cores to the product of the numbers of nodes within two seed cores, designated as B_*f*_ and B_*t*_ is larger or equal to θ, we calculate each pair of seed cores’ value *S*_*wd*_ [Eq. (3)], which is a convex combination of two parts. The first part is the weighted sum of weights of authentic edges; the second part is the percent of the authentic number of edges to the number of maximum possible edges. The two seed cores were then merged into one if the ratio of them is maximal. Repeat the Step 6 until there were no two seed cores that met the criteria as aforementioned.

3 $${S}_{wd}=\alpha \frac{{\sum }_{k=1}^{n}{x}_{k}^{2}}{{\sum }_{k=1}^{n}{x}_{k}}+(1-\alpha )\ast 100\ast n/({B}_{f}\ast {B}_{t}),\alpha \in [0,1]\,{\rm{and}}\,n/({B}_{f}\ast {B}_{t})\ge \theta .$$

Where *x*_*k*_ is the weight of an authentic connection of two nodes in two seed cores, n is the number of authentic connections between the two seed cores, B_*f*_ and B_t_ and are the number of nodes in the two different seed cores. The parameter *α* was used to control the proportion of importance between the sum of all weights of authentic connections and the number of authentic connections.

Step 7. Adding other individual nodes that did not have complete connections to every node in a seed core but had a significant number of connections to each seed core. If the ratio of the number of authentic connections between a candidate node and a seed core to the number of nodes in the seed is larger or equal to **θ**, then we calculated its scoring value, T [Eq. (4)]. The intuition behind Eq. (4) is similar to Eq. (3). We consider both the weighted sum of weights of authentic edges and the percent of the number of authentic edges to the maximum number of possible edges. MSGA added it into the seed core with maximum T. We repeated Step 7, until there are not any candidate nodes can be added into any of the seed cores.

4 $${\rm{T}}=\alpha \frac{{\sum }_{k=1}^{n}{x}_{k}^{2}}{{\sum }_{k=1}^{n}{x}_{k}}+(1-\alpha )\ast 100\ast {{\rm{n}}}_{x}/{{\rm{N}}}_{x},\alpha \in [0,1]and{{\rm{n}}}_{x}/{{\rm{N}}}_{x}\ge \theta .$$

Where *x*_*k*_ is the weight of an authentic connection between the candidate node and one of nodes within the seed core, n_*x*_ is the number of the candidate node’s authentic connections for the seed core, N_*x*_ is the number of nodes in the seed core.

Step 8. Calculate the association scores of each subnetwork and output them in order. After all seed become matured, we remove some subnetworks that the number of nodes is smaller than γ, calculate all the subnetworks’ value M [Eq. (5)], which is also a convex combination of two parts: one is weighted sum of weights of authentic edges, the other is the percent of the number of authentic edges to the number of maximum possible edges, and then sort them by M values in descending order for output.

5 $${\rm{M}}=\alpha \frac{{\sum }_{k=1}^{n}{x}_{k}^{2}}{{\sum }_{k=1}^{n}{x}_{k}}+(1-\alpha )\ast 100\ast {{\rm{d}}}_{x}/[{{\rm{D}}}_{x}\ast ({{\rm{D}}}_{x}-1)],\alpha \in [0,1].$$

Where *x*_*k*_ is the weight of the authentic connection between any two nodes in a subnetwork, d_*x*_ is the number of authentic connections in the subnetwork, D_*x*_ is the number of nodes in the subnetwork. The *α* is a parameter that controls the proportion of importance between the weight of authentic connections and the number of authentic connections in the subnetwork.

### Comparison of the internal association strengths of the subnetworks derived from SSGA, MSGA and Triple-Link Algorithm

The following four cluster validity indices were used to evaluate the subnetworks derived from three algorithms.

Davies-Boulin (DB↓) index has been developed to measure the similarity of subnetworks average intra-subnetwork in comparison with estimates the average of subnetwork similarities based on the ratio of sum of average distances (from the points in a subnetwork to its centroid) to the distance between centroids^17^, which means that for each subnetwork calculate the worst relation index between itself and one of the other subnetworks and finally get an average value. For clustering a data, the best value maybe same, but the worst value must different, so we want to use the index to compare the three algorithms. It is defined as the following formula:6

6 $$DB=\frac{1}{K}\sum _{{c}_{j}\in C}{ma}{{x}}_{{C}_{i}\in C\backslash {C}_{j}}\{\frac{S({C}_{j})+S({C}_{i})}{d({c}_{j},{c}_{i})}\}.$$

7 $$S({C}_{j})=\frac{1}{|{C}_{j}|}\sum _{{x}_{i}\in {C}_{j}}d({x}_{i}-{c}_{j}).$$

Where C contains all subnetworks, K is the number of subnetworks, *C*_*i*_ is the i-th subnetwork, *c*_*i*_ is the center of *C*_*i*_, *d*(*c*_*j*_, *c*_*i*_) is distance between centroids *c*_*j*_ and *c*_*i*_, *x*_*i*_ is the node in subnetwork, |*C*_*j*_| is the number of nodes in the subnetwork *C*_*j*_.

Davies-Bouldin* (DB*↓) estimates the cohesion based on the distance from the points in a subnetwork to its centroid and the separation based on the distance between centroids^18^, which is similar to DB, but has a big difference. Due to the subnetwork of the maximal value of sum of average distance and the subnetwork of the minimal of distance between two centroids may be not the same one, so DB* using another way to test the results. It is defined as the following formula:

8 $$D{B}^{\ast }=\frac{1}{K}\sum _{{C}_{j}\in C}\frac{ma{x}_{{C}_{i}\in C{\rm{\setminus }}{C}_{j}}\{S({C}_{j})+S({C}_{i})\}}{mi{n}_{{C}_{i}\in C{\rm{\setminus }}{C}_{j}}\{d({c}_{j},{c}_{i})\}}.$$

Where C contains all subnetworks, K is the number of subnetworks, *C*_*i*_ is the i-th subnetwork, *c*_*i*_ is the center of *C*_*i*_, *d*(*c*_*j*_, *c*_*i*_) is distance between centroids *c*_*j*_ and *c*_*i*_, the definition of S(*C*_*j*_) is the same as in formula 7. Xie-Beni (XB↓) defines the inter-subnetwork separation as the minimum square distance between subnetwork centers, and the intra-subnetwork compactness as the mean square distance between each node and its subnetwork’s center^19^, which calculates a ratio to test the worst value worst, but the XB is a average value for all genes, nor only the genes in one subnetwork. It is defined as the following formula:

9 $$XB=\frac{{\sum }_{{C}_{i}\in C}{\sum }_{{x}_{j}\in {C}_{i}}d{({x}_{j},{c}_{i})}^{2}}{n\ast mi{n}_{i,j\ne i}d{({c}_{i},{c}_{j})}^{2}}.$$

Where C contains all subnetworks, *C*_*i*_ is the i-th subnetwork, *c*_*i*_ is the center of *C*_*i*_, d is distance, *x*_*i*_ is the node in subnetwork and n is the number of nodes in all the subnetworks.

Xie-Beni *(XB*↓) is the ratio of the maximum average of the squares’ sum in subnetwork to the minimum sum of squares between subnetwork centers^18^, which just find out the worst subnetwork and use it as a final judgement. It is defined as the following formula:

10 $$X{B}^{\ast }=\frac{ma{x}_{{C}_{i}\in C}\{\frac{{\sum }_{j=1}^{{n}_{i}}d{({x}_{j},{c}_{i})}^{2}}{{n}_{i}}\}}{mi{n}_{i,j\ne i}d{({c}_{i},{c}_{j})}^{2}}.$$

﻿﻿﻿Where C contains all subnetworks, *C*_*i*_ is the i-th subnetwork, *x*_*j*_ is the node in subnetwork, *n*_*i*_ is the number of nodes in the subnetwork *C*_*i*_ and *d* is distance﻿. ﻿﻿In this paper, we defined *S*(*C*_*i*_) as the average of spearman Rhos within subnetwork *C*_*i*_, and *d*(*c*_*i*_, *c*_*j*_) as the average of spearman Rhos between subnetwork *C*_*i*_ and *C*_*i*_, and exchange the role of “min” and “max” accordingly. Therefore, larger DB, DB*, XB and XB* values in this paper indicate a better clustering algorithm.

## Electronic supplementary material

Supplemental_information_file_final

Supplementary Tables 3-6
