# Supplementary material for: SSGA and MSGA: two seed-growing algorithms for constructing collaborative subnetworks
Source: Sci Rep. 2017 May 3;7:1446. doi: 10.1038/s41598-017-01556-z (PMC5431152; doi:10.1038/s41598-017-01556-z)
Supplement: Supplementary file 1 — Supplemental_information_file_final [file 41598_2017_1556_MOESM1_ESM.pdf]

SSGA and MSGA: two seed-growing algorithms for constructing collaborative subnetworks

Xiaohui Ji[1, 2], Su Chen[2], Jun Cheng Li[3], Wenping Deng[4], Zhigang Wei[2] and Hairong Wei [4, 5, 6*]
[1]College of Information and Computer Engineering, [2]State Key Lab of Forest Genetics and Breeding, Northeast Forestry University, Harbin, Heilongjiang 150040, People's Republic of China.
[3] Guangdong Key Laboratory for Innovative Development and Utilization of Forest Plant Germplasm, South China Agricultural University, Guangzhou 510642, People's Republic of China
[4] School of Forest Resources and Environmental Science, Michigan Technological University, Houghton, MI 49931, United States of America. [5] Department of Computer Science, Michigan Technological University, Houghton, MI 49931, United States of America. [6]Life Science and Technology Institute, Michigan Technological University, Houghton, MI 49931, United States of America.

* Correspondence author: Hairong Wei (hairong@mtu.edu)

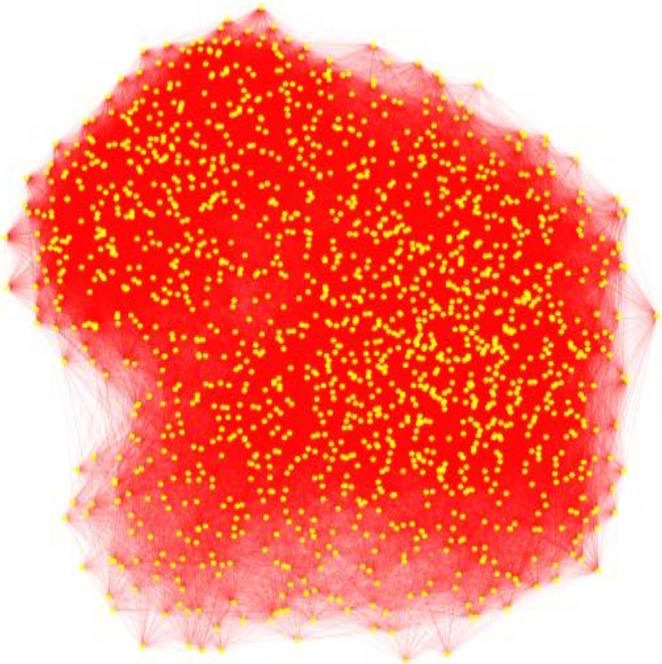

Supplementary Figure 1. The whole collaborative network constructed with Spearman rank correlation for algorithm development. The network was built with 108 normalized microarray chip data described in Nie et al BMC Systems Biology20115:53 DOI: 10.1186/1752-0509-5-53

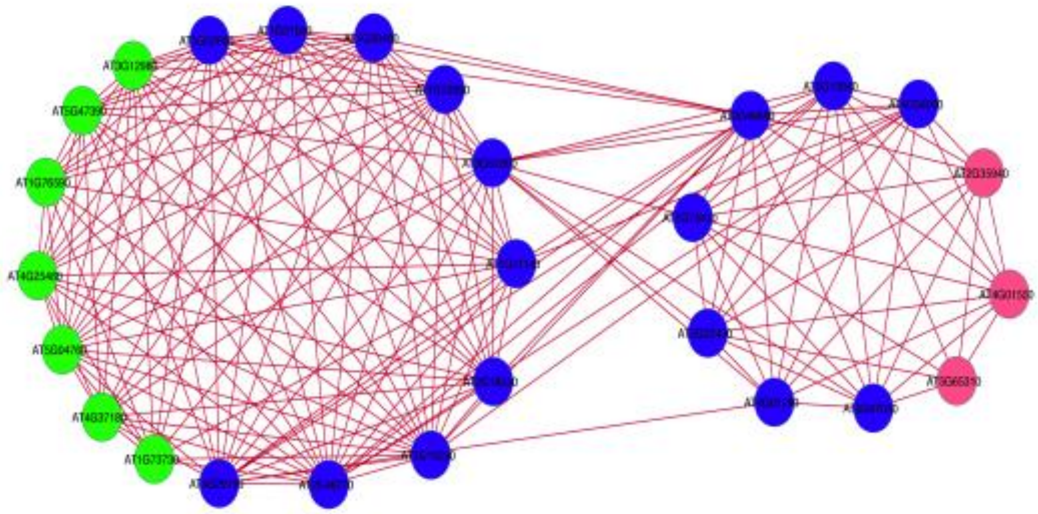

Supplementary Figure 2. Transcription factors (blue nodes) in the Subnetwork 11 derived from Triple-Link algorithm were split into Subnetworks 16 (left) and 18 (right) by SSGA. Green and pink colored nodes are new transcription factors shown up in the Subnetworks 16 and 18 respectively.

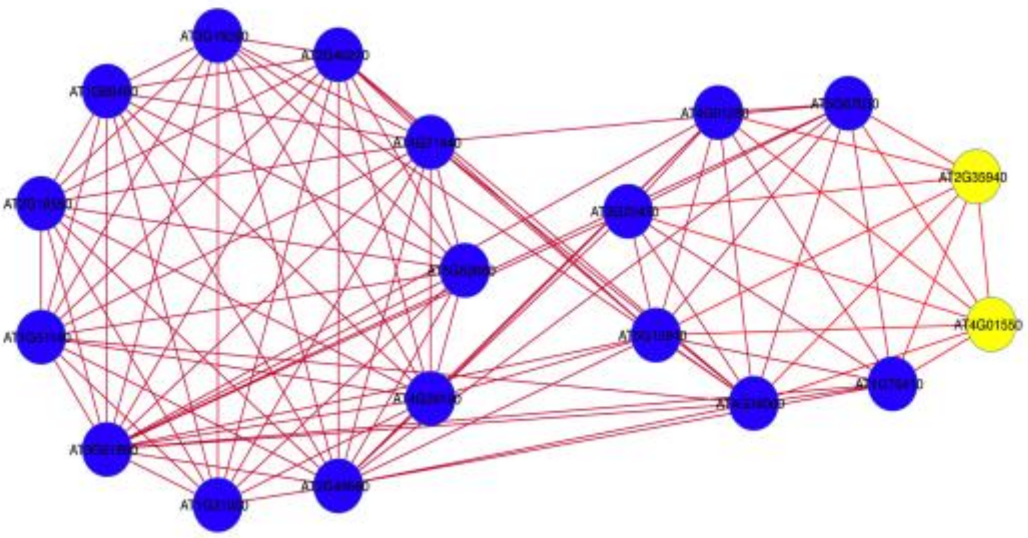

Supplementary Figure 3. Transcription factors (blue nodes) in the Subnetwork 11 derived from Triple-Link algorithm were split into Subnetworks 16 (left) and 8 (right) by MSGA. Yellow colored nodes are new transcription factors shown up in the Subnetwork 8.

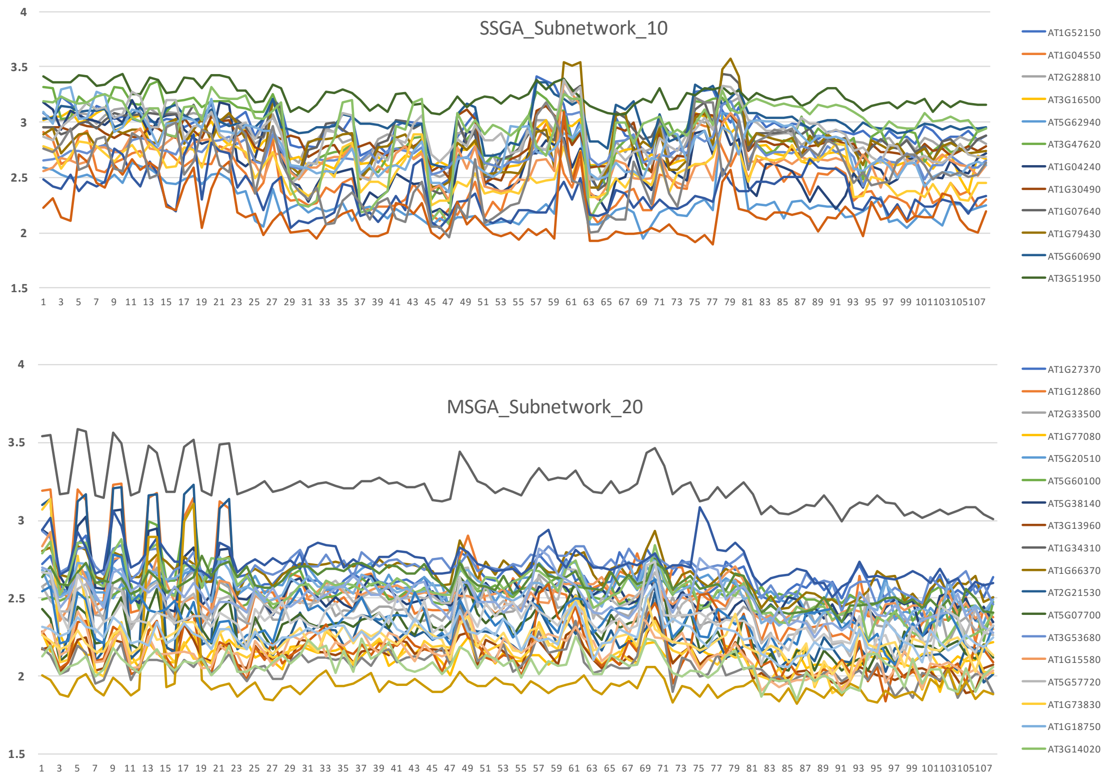

Supplementary Figure 4. The profiles of all genes in two randomly chosen subnetworks, one from SSGA and the other one from MSGA, were shown to display the loosely coordinated TFs within the same subnetworks.

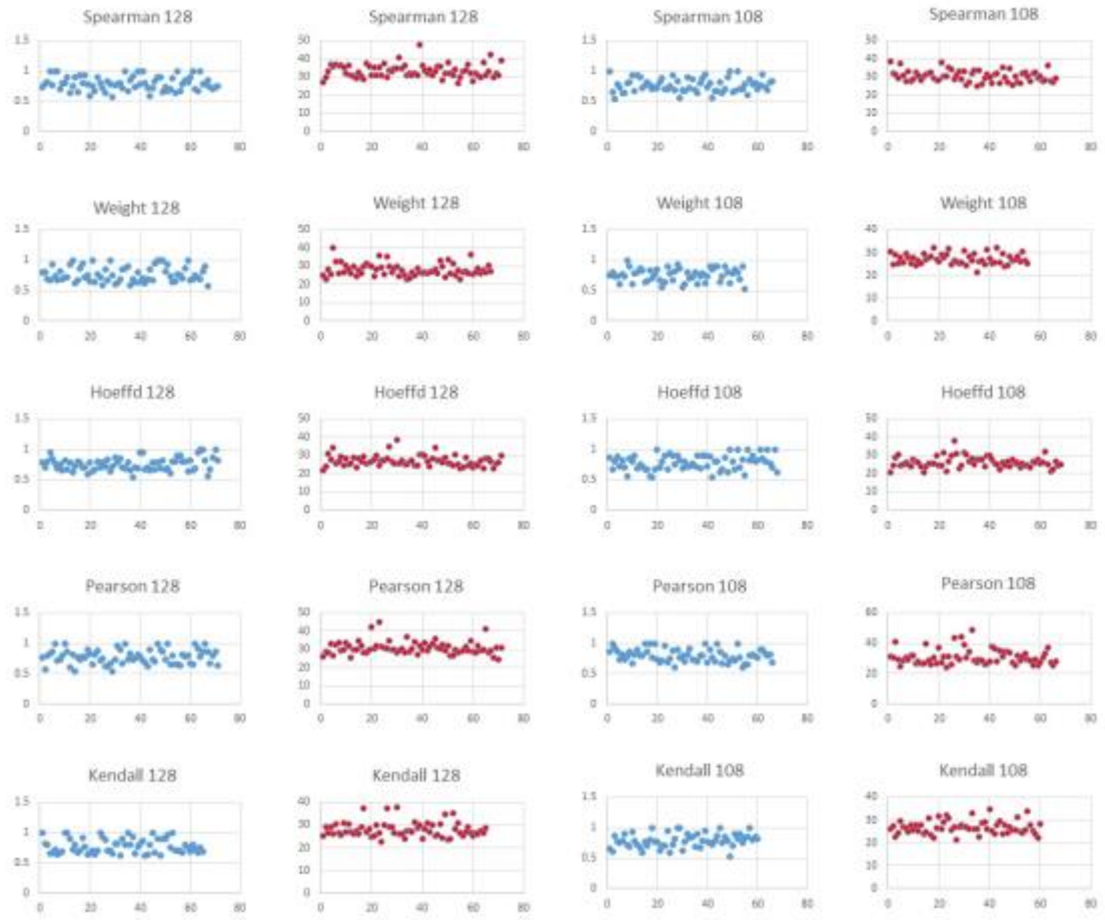

Supplementary Figure 5. The variation of average number of connections per node (blue) and average edge weight (red) within each subnetwork (number of nodes ≥ 5). Each blue point represents an average number of connections between a subnetwork node and all other intra-subnetwork nodes divided by the number of nodes in the subnetwork. Each Red point represents an average edge weight of all edges within a subnetwork. The collaborative networks for decomposition and construction of subnetworks were constructed through a coexpression analysis between each transcription factor and all genomic genes using Spearman rank correlation, Weighted rank correlation, Hoeffding's D measure, Pearson, or Kendall, which was followed by building shared coordination matrices.

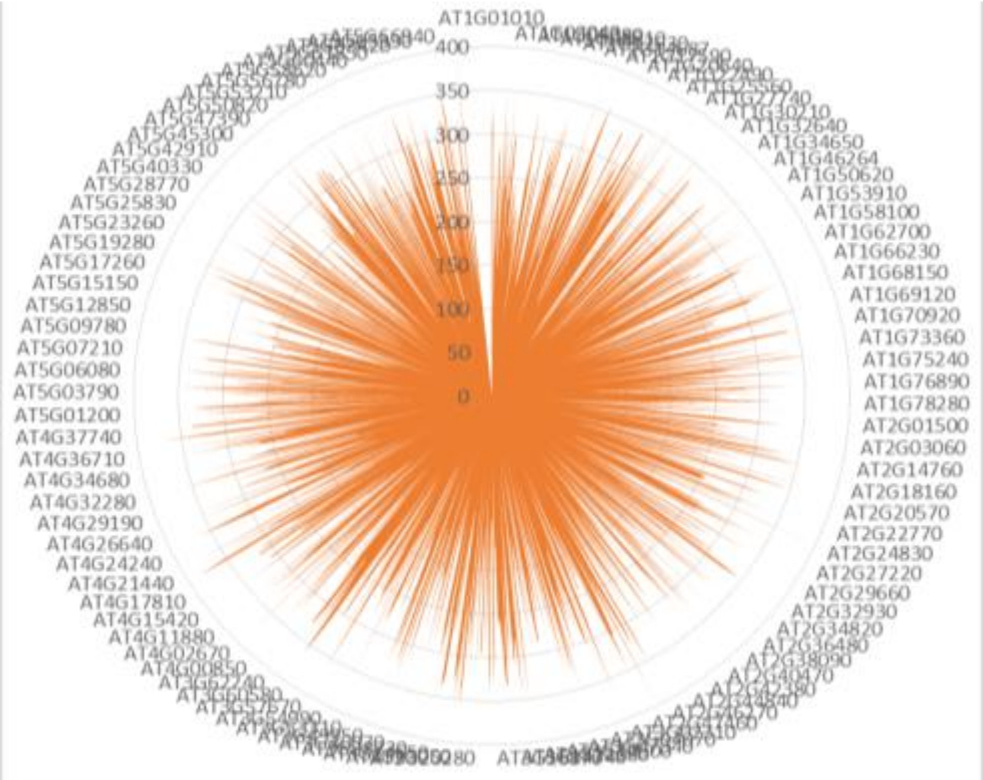

Supplementary Figure 6. The degrees of all regulatory genes in the collaborative network built using Spearman rank correlation and 108 normalized microarray chip data described in Nie et al BMC Systems Biology20115:53 DOI: 10.1186/1752-0509-5-53.

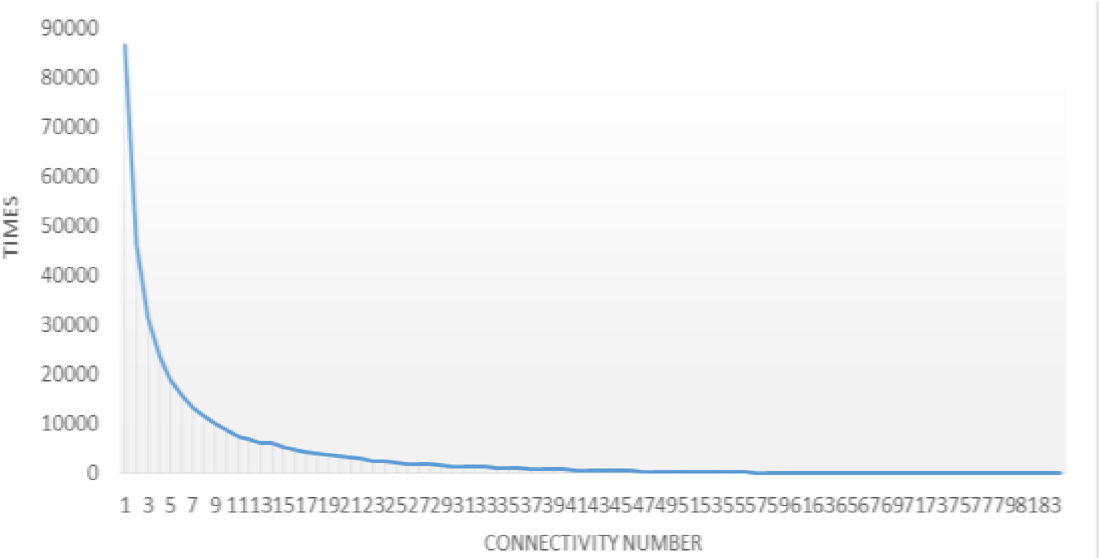

Supplementary Figure 7. The times of connectivity numbers in the collaborative network built using Spearman rank correlation and 108 normalized microarray chip data described in Nie et al BMC Systems Biology20115:53 DOI: 10.1186/1752-0509-5-53.

Supplementary Table 1. The Subnetwork functions of SSGA result.

Gene	Symbol	Description	Evidence
**Subnetwork 1**	**Function: control the root hair growth**		
AT1G27740	RSL4	Postmitotic cell growth in root-hair cells	[1]
AT5G58010	LRL3	Roothairless1	[2]
AT5G19790	RAP2.11	Ethylene response factor controlling root growth	[3]
AT5G25810	TINY	ERF/AP2 TF control cell expansion in root	[4]
AT2G28160	FRU	Regulates iron uptake responses in outer cells of root	[5]
AT1G66470	RHD6	Early root hair formation	[6]
AT1G57560	MYB50	myb domain protein 50	
AT1G30650	WRKY14	WRKY DNA-binding protein 14	[7]
AT5G60120	TOE2	target of early activation tagged (EAT) 2	
**Subnetwork 5**	**Function: control root vascular development, second wall growth development**		
AT1G71930	VND7	Regulates xylem vessel formation	[8]
AT5G12870	MYB46	Target of SND1, control second wall biosynthesis	[9]
AT4G28500	SND2	Vascular cell differentiation	[10]
AT4G00220	LBD30	A central regulator of auxin distribution and signaling in root	[11]
AT1G63910	MYB103	Second wall growth	[12]
AT1G12260	VND4	Switches for protoxylem and metaxylem vessel formation	[13]
AT1G68810	bHLH	Root vascular initial	[14]
AT2G45420	LBD18	Lateral root and tracheary element formation	[15]
AT1G66230	MYB20	Second wall growth	[16]
AT1G73410	MYB54	Second wall growth	[16]
AT1G17950	MYB52	Second wall growth	[16]
AT3G21270	DOF2	Early stages of vascular development	[17]
AT1G01780	PLIM2b	LIM domain-containing protein	
AT1G80730	ZFP1	zinc-finger protein 1	[18]

Supplementary Table 2. The Subnetwork functions of MSGA result.

Gene	Symbol	Description	Evidence
**Subnetwork 2**	**Function: control root cap development and orientation of cell division plane**		
AT2G37260	WRKY44	Differentiation of trichomes and root hairless cells	[19]
AT1G74500	TMO7	Embryonic root initiation	[20]
AT2G30340	LBD13	Expressed in cells at the adaxial base of lateral roots	[21]
AT4G31920	RR10	response regulator 10	[22]
AT2G29060	---	scarecrow transcription factor family protein	
AT5G14750	WER1	WEREWOLF 1	[23]
AT1G33280	NAC015	BRN1, SMB control root cap maturation	[24]
AT4G10350	NAC070	BRN2, SMB control root cap maturation	[24]
AT2G40470	LBD15	Expressed in cells at the adaxial base of lateral roots	[21]
AT5G39820	NAC094	Apical meristem protein, function unknown	[1]
AT1G21340	---	Dof-type zinc finger DNA-binding protein	
AT5G57420	IAA33	IAA is involved in root development	[25]
AT1G26870	FEZ	FEZ and SMB control root stem cells in cap	[26]
AT1G77200	---	DREB subfamily A-4 of ERF/AP2 transcription factor	
AT1G36060	---	Controls Cell Dedifferentiation	[27]
AT1G66350	RGL1	Root epidermal differentiation	[28]
AT1G79580	SMB	FEZ and SMB control root stem cells	[24]
**Subnetwork 4**	**Function: control root cell cycle & growth**		
AT2G22840	GRF1	Growth factor expressed in root	[29]
AT4G37740	GRF2	Growth factor expressed in root	[29]
AT2G36400	GRF3	Growth factor expressed in root	[29]
AT3G01330	E2L2	Cyclin D/retinoblastoma/E2F pathway	[30]
AT5G24330	SDG34	Cell cycle regulation of late G1 to S phase	[31]
AT1G34355	PS1	Parallel spindle 1 involved in meiosis	[32]

SUPPLEMENTARY REFERENCES

1. Yi K, Menand B, Bell E, Dolan L: **A basic helix-loop-helix transcription factor controls cell growth and size in root hairs**. *Nature genetics* 2010, **42**(3):264-U108.
2. Karas B, Amyot L, Johansen C, Sato S, Tabata S, Kawaguchi M, Szczyglowski K: **Conservation of Lotus and Arabidopsis Basic Helix-Loop-Helix Proteins Reveals New Players in Root Hair Development**. *Plant Physiol* 2009, **151**(3):1175-1185.
3. Jung J, Won SY, Suh SC, Kim H, Wing R, Jeong Y, Hwang I, Kim M: **The barley ERF-type transcription factor HvRAF confers enhanced pathogen resistance and salt tolerance in Arabidopsis**. *Planta* 2007, **225**(3):575-588.
4. Wilson K, Long D, Swinburne J, Coupland G: **A dissociation insertion causes a semidominant mutation that increases expression of TINY, an arabidopsis gene related to APETALA2**. *Plant Cell* 1996, **8**(4):659-671.
5. Jakoby M, Wang HY, Reidt W, Weisshaar B, Bauer P: **FRU (BHLH029) is required for induction of iron mobilization genes in Arabidopsis thaliana**. *Febs Lett* 2004, **577**(3):528-534.
6. Menand B, Yi KK, Jouannic S, Hoffmann L, Ryan E, Linstead P, Schaefer DG, Dolan L: **An ancient mechanism controls the development of cells with a rooting function in land plants**. *Science* 2007, **316**(5830):1477-1480.
7. Lee JY, Colinas J, Wang JY, Mace D, Ohler U, Benfey PN: **Transcriptional and posttranscriptional regulation of transcription factor expression in Arabidopsis roots**. *P Natl Acad Sci USA* 2006, **103**(15):6055-6060.
8. Li Z, Omranian N, Neumetzler L, Wang T, Herter T, Usadel B, Demura T, Giavalisco P, Nikoloski Z, Persson S: **A Transcriptional and Metabolic Framework for Secondary Wall Formation in Arabidopsis**. *Plant Physiol* 2016, **172**(2):1334-1351.
9. Zhao Q, Zeng Y, Yin Y, Pu Y, Jackson LA, Engle NL, Martin MZ, Tschaplinski TJ, Ding S-Y, Ragauskas AJ: **Pinoresinol reductase 1 impacts lignin distribution during secondary cell wall biosynthesis in Arabidopsis**. *Phytochemistry* 2015, **112**:170-178.
10. Sakamoto S, Mitsuda N: **Reconstitution of a secondary cell wall in a secondary cell wall-deficient Arabidopsis mutant**. *Plant and Cell Physiology* 2014:pcu208.
11. Bureau M, Rast MI, Illmer J, Simon R: **JAGGED LATERAL ORGAN (JLO) controls auxin dependent patterning during development of the Arabidopsis embryo and root**. *Plant Mol Biol* 2010, **74**(4-5):479-491.
12. Hussey SG, Mizrachi E, Spokevicius AV, Bossinger G, Berger DK, Myburg AA: **SND2, a NAC transcription factor gene, regulates genes involved in secondary cell wall development in Arabidopsis fibres and increases fibre cell area in Eucalyptus**. *Bmc Plant Biol* 2011, **11**.
13. Zhou JL, Zhong RQ, Ye ZH: **Arabidopsis NAC Domain Proteins, VND1 to VND5, Are Transcriptional Regulators of Secondary Wall Biosynthesis in Vessels**. *Plos One* 2014, **9**(8).
14. Ohashi-Ito K, Bergmann DC: **Regulation of the Arabidopsis root vascular initial population by LONESOME HIGHWAY**. *Development* 2007, **134**(16):2959-2968.
15. Lee HW, Kim NY, Lee DJ, Kim J: **LBD18/ASL20 Regulates Lateral Root Formation in Combination with LBD16/ASL18 Downstream of ARF7 and ARF19 in Arabidopsis**. *Plant Physiol* 2009, **151**(3):1377-1389.
16. Zhong RQ, Lee CH, Zhou JL, McCarthy RL, Ye ZH: **A Battery of Transcription Factors Involved in the Regulation of Secondary Cell Wall Biosynthesis in Arabidopsis**. *Plant Cell* 2008, **20**(10):2763-2782.
17. Gardiner J, Sherr I, Scarpella E: **Expression of DOF genes identifies early stages of vascular development in Arabidopsis leaves**. *Int J Dev Biol* 2010, **54**(8-9):1389-1396.
18. Nishii A, Takemura M, Fujita H, Shikata M, Yokota A, Kohchi T: **Characterization of a novel gene encoding a putative single zinc-finger protein, ZIM, expressed**

during the reproductive phase in Arabidopsis thaliana. *Biosci Biotech Bioch* 2000, **64**(7):1402-1409.

19. Bruex A, Kainkaryam RM, Wieckowski Y, Kang YH, Bernhardt C, Xia Y, Zheng XH, Wang JY, Lee MM, Benfey P *et al*: **A Gene Regulatory Network for Root Epidermis Cell Differentiation in Arabidopsis**. *PLoS genetics* 2012, **8**(1).
20. Schlereth A, Moller B, Liu WL, Kientz M, Flipse J, Rademacher EH, Schmid M, Jurgens G, Weijers D: **MONOPTEROS controls embryonic root initiation by regulating a mobile transcription factor**. *Nature* 2010, **464**(7290):913-U128.
21. Shuai B, Reynaga-Pena CG, Springer PS: **The LATERAL ORGAN BOUNDARIES gene defines a novel, plant-specific gene family**. *Plant physiology* 2002, **129**(2):747-761.
22. Street IH, Mathews DE, Yamburkenko MV, Sorooshzadeh A, John RT, Swarup R, Bennett MJ, Kieber JJ, Schaller GE: **Cytokinin acts through the auxin influx carrier AUX1 to regulate cell elongation in the root**. *Development* 2016, **143**(21):3982-3993.
23. Cheng YW, Zhu WJ, Chen YX, Ito S, Asami T, Wang XL: **Brassinosteroids control root epidermal cell fate via direct regulation of a MYB-bHLH-WD40 complex by GSK3-like kinases**. *eLife* 2014, **3**.
24. Bennett T, van den Toorn A, Sanchez-Perez GF, Campilho A, Willemsen V, Snel B, Scheres B: **SOMBRERO, BEARSKIN1, and BEARSKIN2 Regulate Root Cap Maturation in Arabidopsis**. *Plant Cell* 2010, **22**(3):640-654.
25. Kares C, Prinsen E, Van Onckelen H, Otten L: **IAA synthesis and root induction with iaa genes under heat shock promoter control**. *Plant molecular biology* 1990, **15**(2):225-236.
26. Willemsen V, Bauch M, Bennett T, Campilho A, Wolkenfelt H, Xu J, Haseloff J, Scheres B: **The NAC Domain Transcription Factors FEZ and SOMBRERO Control the Orientation of Cell Division Plane in Arabidopsis Root Stem Cells**. *Dev Cell* 2008, **15**(6):913-922.
27. Iwase A, Mitsuda N, Koyama T, Hiratsu K, Kojima M, Arai T, Inoue Y, Seki M, Sakakibara H, Sugimoto K: **The AP2/ERF transcription factor WIND1 controls cell dedifferentiation in Arabidopsis**. *Curr Biol* 2011, **21**(6):508-514.
28. Gan Y, Yu H, Peng J, Broun P: **Genetic and molecular regulation by DELLA proteins of trichome development in Arabidopsis**. *Plant physiology* 2007, **145**(3):1031-1042.
29. Liu JY, Rice JH, Chen NN, Baum TJ, Hewezi T: **Synchronization of Developmental Processes and Defense Signaling by Growth Regulating Transcription Factors**. *PLoS One* 2014, **9**(5).
30. Van Leene J, Hollunder J, Eeckhout D, Persiau G, Van de Slijke E, Stals H, Van Isterdael G, Verkest A, Neirynck S, Buffel Y *et al*: **Targeted interactomics reveals a complex core cell cycle machinery in Arabidopsis thaliana**. *Molecular systems biology* 2010, **6**.
31. Raynaud C, Sozzani R, Glab N, Domenichini S, Perennes C, Cella R, Kondorosi E, Bergounioux C: **Two cell-cycle regulated SET-domain proteins interact with proliferating cell nuclear antigen (PCNA) in Arabidopsis**. *Plant Journal* 2006, **47**(3):395-407.
32. d'Erfurth I, Jolivet S, Froger N, Catrice O, Novatchkova M, Simon M, Jenczewski E, Mercier R: **Mutations in AtPS1(Arabidopsis thaliana Parallel Spindle 1) Lead to the Production of Diploid Pollen Grains**. *PLoS genetics* 2008, **4**(11).
